# Polyamine metabolism is a central determinant of helper T cell lineage fidelity

**DOI:** 10.1016/j.cell.2021.06.007

**Published:** 2021-08-05

**Authors:** Daniel J. Puleston, Francesc Baixauli, David E. Sanin, Joy Edwards-Hicks, Matteo Villa, Agnieszka M. Kabat, Marcin M. Kamiński, Michal Stanckzak, Hauke J. Weiss, Katarzyna M. Grzes, Klara Piletic, Cameron S. Field, Mauro Corrado, Fabian Haessler, Chao Wang, Yaarub Musa, Lena Schimmelpfennig, Lea Flachsmann, Gerhard Mittler, Nir Yosef, Vijay K. Kuchroo, Joerg M. Buescher, Stefan Balabanov, Edward J. Pearce, Douglas R. Green, Erika L. Pearce

**Affiliations:** 1 Max Planck Institute of Immunobiology and Epigenetics, 79108 Freiburg, Germany; 2 The Kennedy Institute of Rheumatology, University of Oxford, Oxford OX3 7FY, UK; 3 Department of Immunology, St. Jude Children’s Research Hospital, Memphis, TN 38105, USA; 4 Evergrande Center for Immunologic Diseases, Harvard Medical School and Brigham and Women’s Hospital, Boston, MA 02115, USA; 5 Broad Institute of MIT and Harvard, Cambridge, MA 02142, USA; 6 Department of Electrical Engineering and Computer Science, University of California, Berkeley, Berkeley, CA 94720, USA; 7 Center for Computational Biology, University of California, Berkeley, Berkeley, CA 94720, USA; 8 Division of Haematology, University Hospital Zurich and University of Zurich, 8091 Zurich, Switzerland; 9 Faculty of Biology, University of Freiburg, 79104 Freiburg, Germany; 10 The Bloomberg∼Kimmel Institute for Cancer Immunotherapy at Johns Hopkins, Johns Hopkins University, Baltimore, MD, USA

**Keywords:** polyamines, hypusine, T cells, immunometabolism, immunity, eIF5A, metabolism

## Abstract

Polyamine synthesis represents one of the most profound metabolic changes during T cell activation, but the biological implications of this are scarcely known. Here, we show that polyamine metabolism is a fundamental process governing the ability of CD4^+^ helper T cells (T_H_) to polarize into different functional fates. Deficiency in ornithine decarboxylase, a crucial enzyme for polyamine synthesis, results in a severe failure of CD4^+^ T cells to adopt correct subset specification, underscored by ectopic expression of multiple cytokines and lineage-defining transcription factors across T_H_ cell subsets. Polyamines control T_H_ differentiation by providing substrates for deoxyhypusine synthase, which synthesizes the amino acid hypusine, and mice in which T cells are deficient for hypusine develop severe intestinal inflammatory disease. Polyamine-hypusine deficiency caused widespread epigenetic remodeling driven by alterations in histone acetylation and a re-wired tricarboxylic acid (TCA) cycle. Thus, polyamine metabolism is critical for maintaining the epigenome to focus T_H_ cell subset fidelity.

## Highlights

• Polyamines govern the ability of CD4^+^ T cells to differentiate into distinct subsets
• Through the synthesis of hypusine, polyamines direct the T cell epigenome
• Loss of polyamines or hypusine perturbs the TCA cycle and histone acetylation
• HAT deletion restores T_H_ differentiation in polyamine-deficient cells

## Graphical abstract

**Figure:**
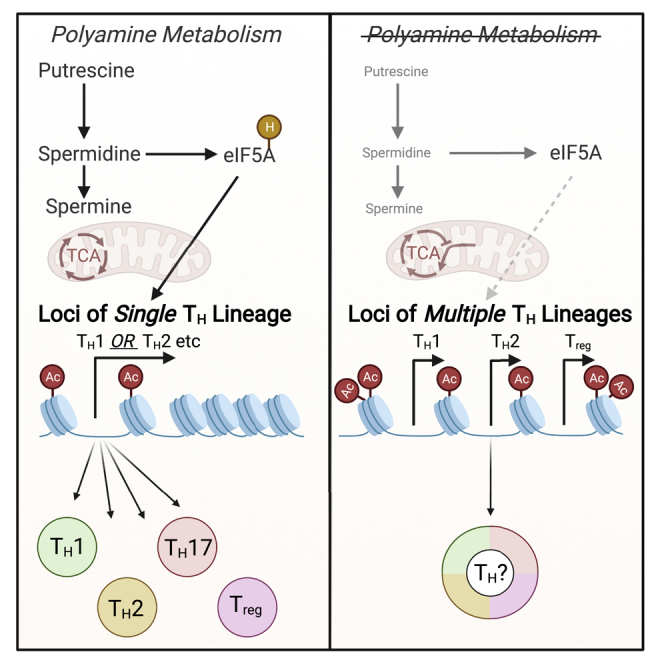

## Teaser

The ability of CD4^+^ T helper cells to develop into specified functional subsets depends upon polyamine-driven metabolic and epigenetic regulation.

## Introduction

Upon activation, T cells proliferate to form effector cells that mediate immunity. For CD4^+^ helper T (T_H_) cells, this clonal expansion is linked to their differentiation into distinct subsets with specialized functions, which are critical for controlling pathogens and maintaining tissue homeostasis. Three major subsets of effector CD4^+^ T_H_ cells are T_H_1, T_H_2, and T_H_17 cells. These cells differentiate from naive T cells in response to signals from antigen presenting cells during activation and local microenvironmental cues. The functional specialization of T_H_ cells is conferred by the expression of T cell subset-specific transcription factors (TFs) that coordinate genetic programs to direct production of signature cytokines and surface molecules mediating interactions with other cells (Murphy and Stockinger, 2010). In a simplified overview, T_H_1 cells express the TF T-bet and the cytokine interferon (IFN)-γ and mediate responses to intracellular pathogens. T_H_2 cells express GATA3 and interleukin (IL)-4 and control helminth infections. T_H_17 cells synthesize RORγt and IL-17 and limit extracellular bacteria and fungi, particularly at mucosal surfaces (Kanno et al., 2012). A fourth subset of T_H_ cell, regulatory T cells (T_regs_), modulates immunity by dampening effector T cell activation and proliferation and expresses the TF Foxp3 (Fontenot et al., 2003). This partitioning of the CD4^+^ T cell response, such that pathogens drive distinct T_H_ effector programs, necessitates that faithful T_H_ differentiation is essential to mount an optimal immune response to any given threat.

Metabolic reprograming is critical for T cell activation and differentiation (Buck et al., 2015). Older reports have shown that polyamine synthesis is a hallmark of T cell activation and proliferation (Bowlin et al., 1987; Kay and Pegg, 1973; Schall et al., 1991; Scott et al., 1985) but its functional implications have not been widely investigated. In mammalian cells, the pool of polyamines comprises putrescine, spermidine, and spermine. During polyamine synthesis, the amino acid ornithine is converted to putrescine by the rate limiting enzyme ornithine decarboxylase (ODC). Putrescine can then be metabolized to spermidine and eventually spermine.

In this study, we investigate the role of polyamine metabolism in CD4^+^ T cell differentiation and function. We show that loss of polyamine synthesis leads to profound changes in the ability of CD4^+^ T cells to dependably differentiate into functionally distinct subsets. Our data suggest the importance of polyamine metabolism in directing T_H_ subset specification lies in the role of spermidine acting as a substrate for the synthesis of the amino acid hypusine. Deletion of ODC, or the enzymes responsible for hypusine synthesis, results in dramatic epigenomic changes, driven by enhanced histone acetylation. These data place the polyamine-hypusine axis in a position of central importance in CD4^+^ T cell differentiation.

## Results

### Polyamine biosynthesis is dynamically regulated in CD4^+^ T cells

**Figure 1:**
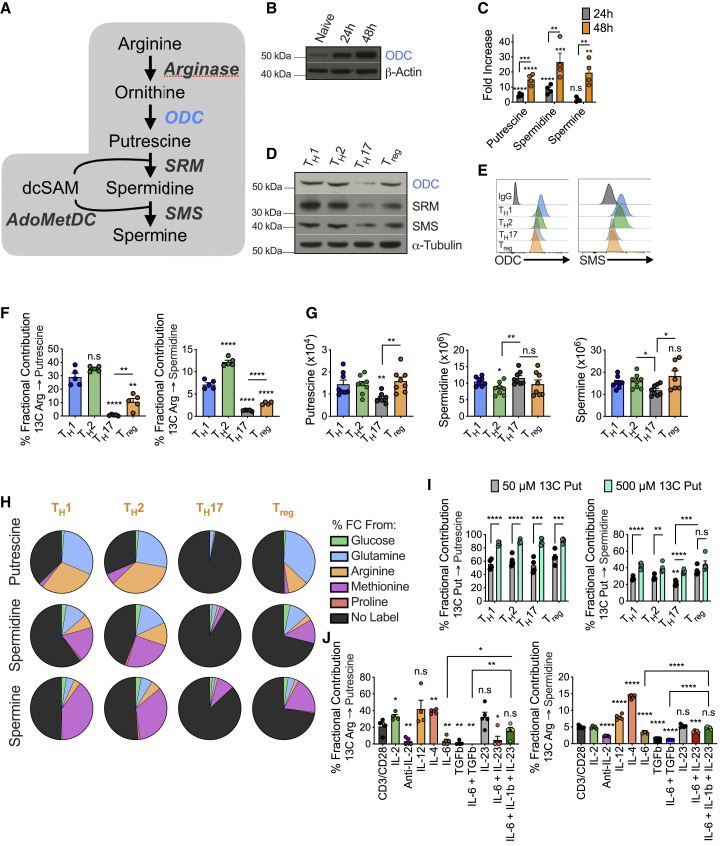
Polyamine metabolism is dynamically regulated in CD4^+^ T cells and directed by cytokines (A) Polyamine (PA) synthesis pathway. (B) Immunoblot of WT naive CD4^+^ T cells activated for indicated time or rested overnight in 10 ng/mL IL-7, representative of 3 biological replicates. (C) Mass spectrometry (MS) analysis of PA levels in CD4^+^ T cells post-activation. Fold increase versus CD4^+^ T cells rested overnight in IL-7. (D) Immunoblot of PA enzymes in CD4^+^ T cells under stated condition day 4 post-activation, representative of 3 biological replicates. (E) ODC and SMS levels in CD4^+^ T_H_ cells by flow cytometry (FC) 96 h post-activation. (F) Naive CD4^+^ T cells polarized for 72 h then exposed to 1.1 mM ^13^C arginine for 24 h. (G) MS analysis of PA levels in naive CD4^+^ T_H_ cells polarized for 96 h. (H) Naive CD4^+^ T cells differentiated under various T_H_ conditions. On day 3, cells were treated for 24 h in the labeled substrates stated and tracing into PAs was assessed by MS. (I) Naive CD4^+^ T cells polarized into T_H_ subsets. After 48 h, cells were exposed to ^13^C putrescine for a further 48 h. (J) Naive CD4^+^ T cells activated in the presence of the defined cytokine(s) and/or blocking Ab. After 72 h, cells were treated with 1.1 mM ^13^C arginine for 24 h. ^13^C arginine in PAs assessed by MS. All data are mean ± SEM (p^∗^ < 0.05, p^∗∗^ < 0.005, p^∗∗∗^ < 0.0005, p^∗∗∗∗^ < 0.00005). Data are representative of 2 (C, D, F, and G), 3 (E), 1–2 (H), or 1 experiment (I and J). Asterisks without line bar denote statistical significance relative to T_H_1 cells (F, G, and I) or to CD3/CD28 condition (J). See also Figure S1.

**Figure S1:**
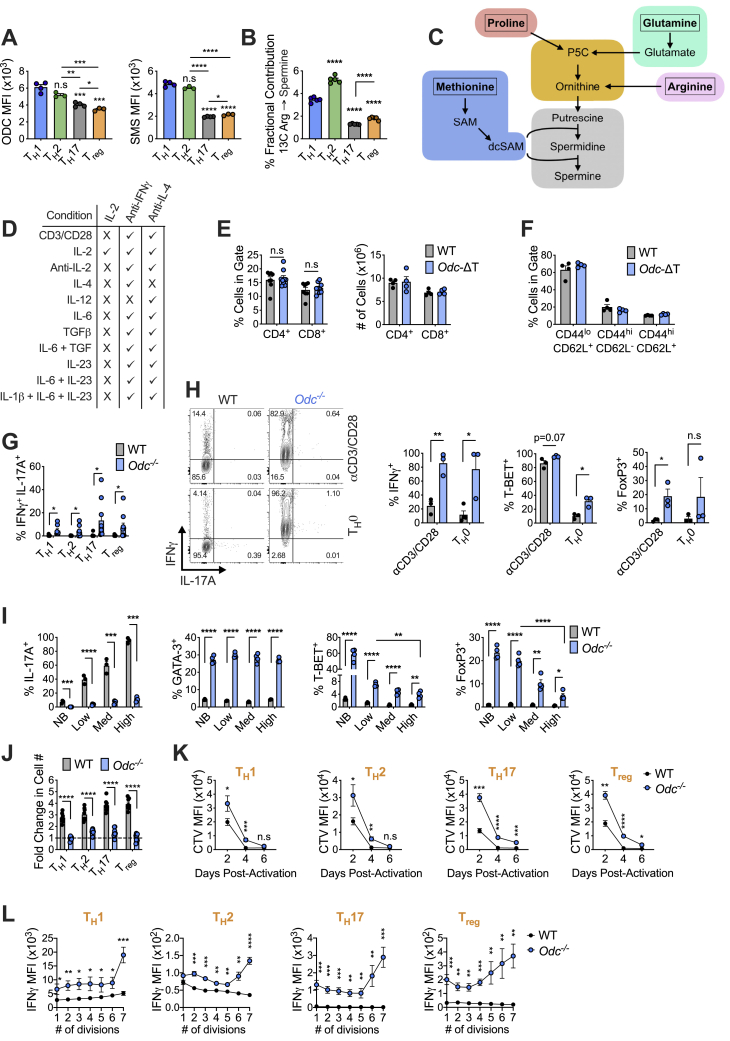
*Odc*^−/−^ CD4^+^ T cells exhibit dysregulated T_H_ lineage commitment and proliferation, related to Figures 1 and 2 (A) Expression of ODC and SMS in CD4^+^ T_H_ cells on day 4 post-activation by flow cytometry (FC). Asterixis without line bar denote statistical significance relative to T_H_1 cells. (B) Naive CD4^+^ T cells isolated from spleen and polarized under different T_H_ conditions for 3 days and then re-plated in fresh T_H_ polarizing media containing 1.1 mM ^13^C arginine for 24 hours. Asterixis without line bar denote statistical significance relative to T_H_1 cells. (C) Schematic depicting potential substrates for polyamine synthesis. (D) Naive CD4^+^ T cells were polarized in the stated cytokines and blocking antibodies. After 72h, cells were replated into fresh media containing fresh cytokines and blocking antibodies and cultured for 24 hours with 1.1 mM ^13^C arginine. Table indicates combinations of cytokines with IL-2 and IFN-γ/IL-4 blocking antibodies. (E) Frequency/absolute numbers of T cells and (F) frequency of naive and memory CD4^+^ T cells in the spleen of 8 week old *Odc*-ΔT mice and their WT littermates. (G) Naive WT and *Odc*^−/−^ CD4^+^ T cells were activated for 4 days in the indicated T_H_ polarizing condition. Cytokine expression after 5 hour PMA/iono restim. (H) Naive WT and *Odc*^−/−^ CD4^+^ T cells activated with anti-CD3/CD28 ± anti-IFN-γ/IL-4 (T_H_0) were assayed for the indicated cytokine and transcription factor by FC 4 days post-activation. Representative contour plots shown. (I) Naive WT and *Odc*^−/−^ CD4^+^ T cells were polarized under T_H_17 conditions ± varying concentrations (low, medium, high, see methods) of blocking antibodies. After 4 days, expression of the indicated cytokine or transcription factors was assessed by FC (NB = no blocking antibody). (J) Fold change in the number of WT and *Odc*^*−/−*^ T_H_ cells between day 0 and day 4 of *in vitro* culture. (K) Naive WT and *Odc*^*−/−*^ CD4^+^ T cells were stained with cell trace violet (CTV) proliferation dye and then polarized into different T_H_ subsets and assessed for CTV levels on the indicated day. (L) Naive WT and *Odc*^*−/−*^ CD4^+^ T cells stained with CTV, polarized and then assessed for IFN-γ after restimulation with PMA/ionomycin on day 4. All data are mean ± SEM (p^∗^ < 0.05, p^∗∗^ < 0.005, p^∗∗∗^ < 0.0005, p^∗∗∗∗^ < 0.00005). Representative of 3 (A, E-F,I), 2 (B,H, J-L), or 5 (G) experiments.

Polyamine metabolism requires ODC (Figure 1A). ODC expression and intracellular polyamines increased after CD4^+^ T cell activation (Figures 1B and 1C). We also questioned if polyamine synthesis was active in differentiated T_H_ subsets. We isolated naive CD4^+^ T cells from C57BL/6 mice and polarized them into T_H_1, T_H_2, T_H_17, and T_reg_ cell subsets (Zhu et al., 2010), then assessed ODC, spermidine synthase (SRM), and spermine synthase (SMS) expression. All three enzymes were expressed across T_H_ subsets, although levels were reduced in T_H_17 and T_regs_ relative to T_H_1 and T_H_2 cells (Figures 1D, 1E, and S1A). Arginine is a major substrate for polyamine synthesis, and we exposed cells to ^13^C arginine to assess its movement through polyamines in T_H_ subsets. Although ^13^C arginine accumulated in polyamines in T_H_1 and T_H_2 cells, this accumulation was significantly lower in T_regs_ and virtually absent in T_H_17 cells (Figures 1F and S1B). However, we observed similar polyamine abundance across T_H_ subsets, except in T_H_17 cells, which had lower putrescine and spermine (Figure 1G). These data imply that polyamine synthesis is active in T_H_ cells, but some subsets, particularly T_H_17 cells and T_regs_, may use substrates other than arginine for polyamines.

Glutamine, arginine, and proline are also substrates for polyamine synthesis, whereas methionine acts as a substrate for spermidine and spermine (Figure S1C). We polarized naive CD4^+^ T cells into T_H_1, T_H_2, T_H_17, and T_regs_ and exposed them to ^13^C-glucose, ^13^C-glutamine, ^13^C-arginine, ^13^C-proline, or ^15^N-methionine for 24 h. Putrescine was synthesized equally from arginine and glutamine in T_H_1 and T_H_2 cells, whereas glutamine was the dominant substrate in T_regs_. T_H_17 cells did not utilize any of these substrates (Figure 1H). For spermidine, methionine, arginine, and glutamine were the dominant substrates compared to just methionine and glutamine in T_regs_. Only a fraction of these substrates contributed to spermidine in T_H_17 cells (Figure 1H). Methionine was the main metabolite utilized for spermine synthesis across T_H_ subsets. Glucose and proline were not significant substrates for polyamine synthesis in CD4^+^ T_H_ cells (Figure 1H). These data suggest that T_H_17, and to a lesser extent T_regs_, may exhibit diminished flux through the polyamine pathway relative to T_H_1 and T_H_2 cells.

Because the polyamine pool remained relatively consistent across T_H_ subsets (Figure 1G), despite disparities in metabolic flux and substrate choice (Figure 1H), we questioned if exogenous uptake of polyamines contributes to intracellular polyamine levels. We assessed the fraction of intracellular putrescine and spermidine derived from exogenous ^13^C putrescine in T_H_ cells. When cells were exposed to 500 μM ^13^C putrescine, ∼90% and 40% of the putrescine and spermidine pool, respectively, derived from exogenous putrescine (Figure 1I). Even at a 10-fold lower concentration, exogenous putrescine contributed ∼50% and 30% to the putrescine and spermidine pool, respectively. All T_H_ subsets had an equal ability to acquire putrescine (Figure 1I). Therefore, polyamine influx from the microenvironment may contribute significantly to intracellular polyamine levels. This could be particularly important for T_H_17 cells and T_regs_, which may have lower polyamine synthesis.

To test if cytokines control polyamine metabolism, we cultured CD4^+^ T cells with ^13^C arginine and combinations of cytokines and blocking antibodies (Figure S1D). Using CD4^+^ T cells activated only with anti-CD3/CD28 and treated with IFN-γ and IL-4 blocking antibodies as a baseline, we found that IL-2, IL-12, or IL-4 enhanced arginine flux into putrescine and spermidine, while this was diminished in cells treated with IL-6, or transforming growth factor β (TGF-β), or both (Figure 1J). The negative impact of IL-6 on polyamine metabolism was reversed when cells were additionally treated with IL-23 and IL-1β (Figure 1J), conditions associated with pathogenic T_H_17 cell development, suggesting that polyamine metabolism may influence the balance between pathogenic and non-pathogenic T_H_17 states. These data confirm that immune factors in the local milieu regulate CD4^+^ T_H_ cell polyamine metabolism and suggest a role for polyamine synthesis in their differentiation.

### Polyamine biosynthesis via *Odc* regulates CD4^+^ T_H_ cell subset fidelity

**Figure 2:**
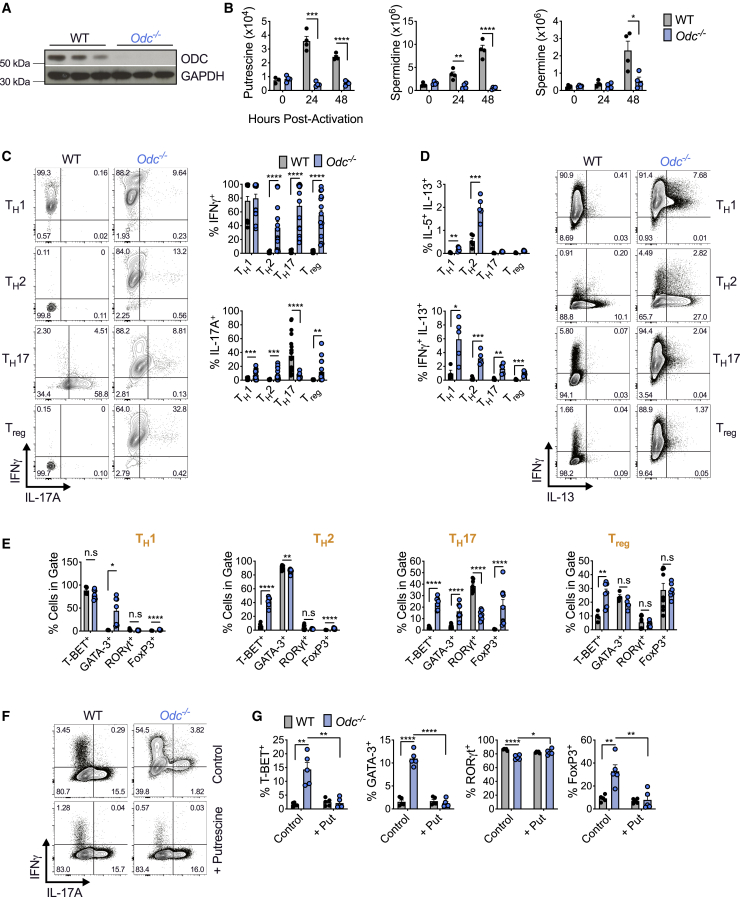
Polyamine biosynthesis via *Odc* regulates CD4^+^ T helper subset fidelity (A) Immunoblot of naive CD4^+^ T cells isolated from WT and *Odc*-ΔT mice 48 h post-activation. (B) MS analysis of PAs in WT and *Odc*^−/−^ CD4^+^ T cells during activation. (C and D) Intracellular cytokines analyzed in WT and *Odc*^−/−^ naive CD4^+^ T cells activated for 96 h in indicated T_H_ condition. Representative contour plots shown. (E) WT and *Odc*^−/−^ CD4^+^ T cells assessed for TF expression on day 4. (F and G) WT and *Odc*^−/−^ naive CD4^+^ T cells activated under T_H_17 cell conditions ± 250 μM putrescine for 96 h. Representative contour plots are shown. All data are mean ± SEM (p^∗^ < 0.05, p^∗∗^ < 0.005, p^∗∗∗^ < 0.0005, p^∗∗∗∗^ < 0.00005). (B, F, and G) Representative of 2, (C) of 3, and (D and E) of 5 experiments. See also Figures S1 and S2.

To examine polyamine metabolism in CD4^+^ T_H_ differentiation, we bred mice with loxP flanked exons 9–11 of *Odc* with mice expressing *CD4*^*cre*^, to generate mice with *Odc* specifically deleted in T cells (*Odc*-ΔT mice). Control mice were absent for cre recombinase. ODC was deleted in T cells from *Odc*-ΔT mice (Figure 2A). *Odc*-ΔT mice displayed comparable CD4^+^ and CD8^+^ T cell frequencies and numbers in spleen, and naive and memory cells within the CD4^+^ T cell compartment, relative to wild-type (WT) littermates (Figures S1E and S1F). Importantly, *Odc*^*−*/*−*^ CD4^+^ T cells had reduced intracellular polyamine levels after activation *in vitro* (Figure 2B). We sorted naive CD4^+^ T cells from *Odc*-ΔT mice and polarized them into T_H_ subsets *in vitro*. After 4 days, we restimulated cells and measured intracellular cytokines. Irrespective of polarizing condition, *Odc*^−/−^ T cells displayed elevated levels of the hallmark T_H_1 cytokine IFN-γ and an increased frequency of cells producing both IFN-γ and IL-17A, the canonical T_H_17 cytokine (Figures 2C and S1G). *Odc*^−/−^ T_H_1 and T_H_2 cells had an increased frequency of cells co-producing the T_H_2 cytokines IL-5 and IL-13 (Figure 2D), and remarkably, an increase in cells co-producing IFN-γ and IL-13 across all *Odc*^−/−^ T_H_ subsets (Figure 2D). Although IL-17A expression increased in T_H_1, T_H_2, and T_reg_ cultures, it decreased under T_H_17 conditions (Figure 2C). Thus, in the absence of *Odc*, T_H_ cells express non-canonical cytokines in a highly dysregulated manner, even when activated in polarizing conditions that normally faithfully direct T_H_ cell subset specification.

Next, we measured lineage-specific TFs in WT and *Odc*^*−*/−^ CD4^+^ T cells under different polarizing conditions. T-bet, the T_H_1 TF (Szabo et al., 2000), was aberrantly expressed in *Odc*^−/−^ T_H_2, T_H_17, and T_reg_ cells, whereas GATA3, the T_H_2 TF, was increased in T_H_1 and T_H_17 cells (Figure 2E). *Odc* deletion also induced the abnormal expression of Foxp3, the T_reg_ cell TF, under T_H_17 conditions (Figure 2E). *Odc*^−/−^ CD4^+^ T cells not exposed to polarizing cytokines, i.e., activated solely with anti-CD3/CD28 plus IL-2, or with the addition of IFN-γ and IL-4 blocking antibodies (T_H_0), also displayed significantly increased IFN-γ, T-bet, and Foxp3 (Figure S1H). The simultaneous expression of multiple lineage-defining TFs and cytokines in polyamine-deficient T_H_ cells implies that polyamine metabolism is central to correct lineage commitment.

**Figure S2:**
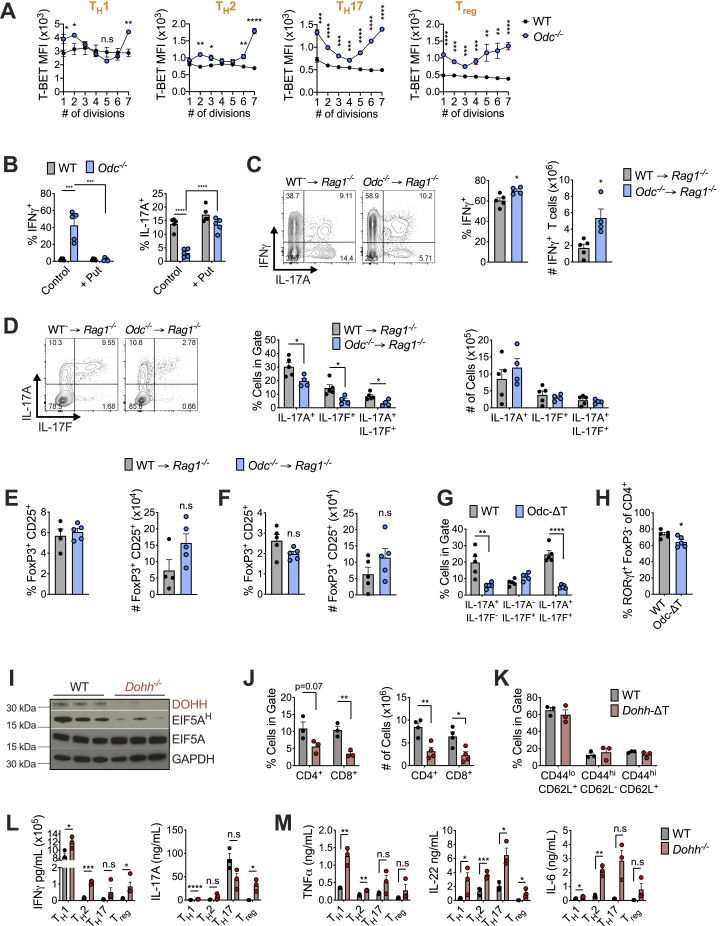
*Odc*^*−/−*^ CD4^+^ T cells display altered effector differentiation *in vivo*, related to Figures 2, 3, and 5 (A) Naive WT and *Odc*^*−/−*^ CD4^+^ T cells stained with CTV, polarized and then assessed for T-Bet expression on day 4. (B) WT and *Odc*^−/−^ naive CD4^+^ T cells activated under T_H_17 cell polarizing conditions ± 250 μM putrescine for 96h. Intracellular cytokine was assessed post re-stimulation with PMA/ionomycin. (C, D) 4x10^5^ WT or *Odc*^*−/−*^ naive (CD45RB^hi^ CD25^-^ CD44^lo^ CD62L^hi^) CD4^+^ T cells were adoptively transferred into *Rag1*^*−/−*^ mice. On day 37, the frequency and number of CD4^+^ T cells in the MLN expressing the indicated cytokine was analyzed following 4 hours *ex vivo* re-stimulation with PMA/ionomycin. (E) Frequency and number of T_regs_ in colon and (F) MLN (day 37 post-transfer) from *Rag1*^*−/−*^ mice that received either WT or *Odc*^*−/−*^ CD4^+^ T cells. (G, H) WT and *Odc*-ΔT mice were treated 50 μg anti-CD3 antibody by i.p injection on day 0, day 2, and day 4 and sacrificed 4h after the last injection. The frequency of CD4^+^ T cells expressing indicated (G) cytokine and (H) TF was assessed from the small intestine by FC. (I) Naive WT and *Dohh*^−/−^ CD4^+^ T cells activated with anti-CD3/28 for 48h were assessed for the indicated proteins by immunoblot. (J) Frequency and absolute numbers of T cells and (K) frequency of naive and memory CD4^+^ T cells in the spleen of 8 week old *Dohh*-ΔT mice and their WT littermates. (L, M) WT and *Dohh*^−/−^ naive CD4^+^ T cells were activated for 96h under T_H_ cell polarizing conditions. Culture supernatent was analyzed for presence of denoted cytokine by cytokine bead array. All data are mean ± SEM (p^∗^ < 0.05, p^∗∗^ < 0.005, p^∗∗∗^ < 0.0005, p^∗∗∗∗^ < 0.00005). (A, C-F, L-M) is representative of 2 experiments, (B,G-H, J-K) Representative of 3 experiments.

To determine if *Odc*^−/−^ CD4^+^ T cell failure to correctly identify a T_H_ lineage was exacerbated by dysregulated cytokines feeding back in an autocrine manner, we exposed T_H_17 cells to increasing concentrations of cytokine blocking antibodies. Simultaneously blocking IL-2, IFN-γ, IL-4, IL-5, and IL-13 enhanced IL-17A production in WT T_H_17 cells, but did not restore IL-17A in *Odc*^−/−^ T_H_17 cells (Figure S1I). Cytokine blocking did not correct aberrant *Odc*^−/−^ T_H_17 cell GATA expression, but reduced T-bet and Foxp3, although not to WT levels (Figure S1I). Loss of polyamine synthesis compromises T cell proliferation (Wang et al., 2011). Fewer *Odc*^−/−^ T cells were present at the end of *in vitro* culture relative to WT cells across T_H_ subsets (Figure S1J), although *Odc* deletion delayed rather than prevented proliferation (Figure S1K). IFN-γ and T-bet were dysregulated in all *Odc*^−/−^ T_H_ subsets regardless of cell division (Figures S1L and S2A). Subset specification defects were exacerbated with increasing cell division (Figures S1L and S2A), suggesting that defective proliferation in polyamine-deficient T cells does not underlie their inability to successfully identify a T_H_ fate.

To confirm that defective putrescine synthesis underlies the phenotype of *Odc*^−/−^ T cells, we treated cells with exogenous putrescine. Putrescine addition restored the ectopic expression of cytokines and TFs of *Odc*^−/−^ T_H_17 cells to WT levels (Figures 2F, 2G, and S2B).

### Purified naive *Odc*^−/−^ CD4^+^ T cells are highly colitogenic in a T cell transfer model of colitis

**Figure 3:**
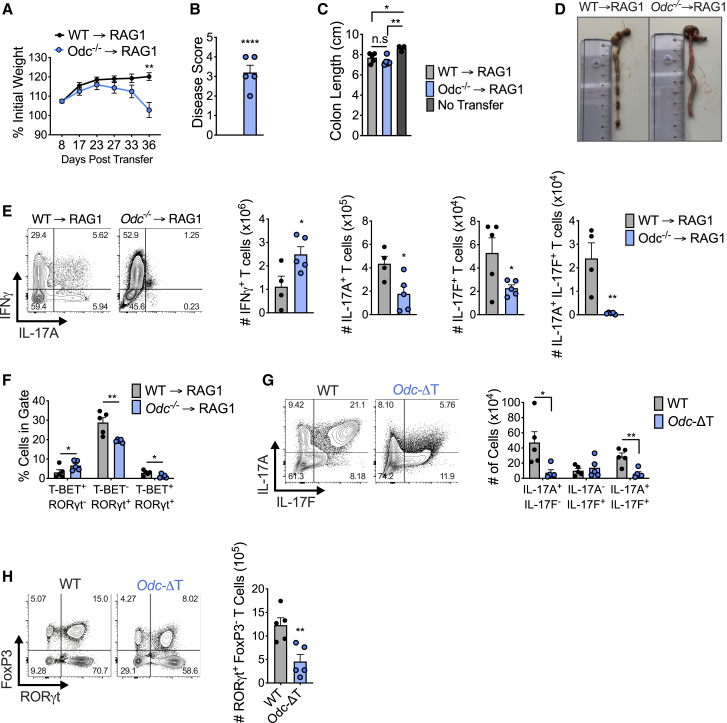
Naive *Odc*^−/−^ CD4^+^ T cells are highly inflammatory in a T cell transfer model of colitis (A) WT or *Odc*^−/−^ naive CD4^+^ T cells were adoptively transferred into *Rag1*^−/−^ mice and weight loss tracked. (B) Disease score (see STAR Methods) assessed in *Rag1*^−/−^ recipients after WT or *Odc*^−/−^ naive CD4^+^ T cell transfer. (C and D) Colon length (C) and representative colon images (D) from *Rag1*^−/−^ mice following WT or *Odc*^−/−^ naive CD4^+^ T cell transfer. (E) Number of CD4^+^ T cells expressing indicated cytokines from colon, representative contour plots shown. (F) Frequency of colonic CD4^+^ T cells from *Rag1*^−/−^ recipient mice expressing indicated TF(s). (G) WT and *Odc*-ΔT mice injected intraperitoneally with 50 μg CD3 monoclonal antibody on days 0, 2, and 4 and sacrificed 4 h after the last injection. Number of CD4^+^ T cells expressing indicated cytokine was assessed from the small intestine (SI) by FC, representative contour plots are shown. (H) CD4^+^ T cells from the SI of WT and *Odc*-ΔT mice, treated as in (G), assessed for the indicated TF. All data are mean ± SEM (p^∗^ < 0.05, p^∗∗^ < 0.005, p^∗∗∗^ < 0.0005, p^∗∗∗∗^ < 0.00005). (A–G) Representative of 2 experiments. See also Figure S2.

*Odc*-ΔT mice appeared normal in terms of health and peripheral T cell numbers in steady state (Figure S1E), but *in vitro*-activated *Odc*^−/−^ T cells displayed profound dysregulation (Figures 2C–2E). To explore this *in vivo*, we used a mouse model of inflammatory bowel disease where transfer of naive CD4^+^ T cells into RAG1-deficient mice, which lack their own lymphocytes, drives colitis (Powrie et al., 1993). We transferred 4 × 10^5^ naive CD4^+^ T cells from WT and *Odc*-ΔT mice into *Rag1*^−/−^ recipient mice. After 3 weeks, *Rag1*^−/−^ mice that received *Odc*^−/−^ T cells began losing weight and declined until 36 days post T cell transfer when the experiment was terminated due to weight loss and diarrhea (Figures 3A and 3B). Although colon length was comparable between mice that received either WT (WT → *Rag1*^−/−^) or *Odc*^−/−^ T cells (*Odc*^−/−^ → *Rag1*^−/−^) (Figure 3C), only mice containing *Odc*^−/−^ T cells showed macroscopic signs of inflammation, characterized by colonic wall thickening (Figure 3D).

We found increased number and frequency of T cells expressing IFN-γ and reduced number and frequency of IL-17A-, IL-17F,- and IL-17A/IL-17F-expressing cells in the colon and mesenteric lymph nodes (MLN) *of Odc*^−/−^ → *Rag1*^−/−^ recipient mice relative to WT → *Rag1*^−/−^ controls (Figure 3E and S2C). A higher frequency of T cells expressed T-bet in the colon of *Odc*^−/−^ → *Rag1*^−/−^ mice, whereas the frequency of T cells expressing RORγt was reduced (Figure 3F). The frequency and number of T cells expressing Foxp3 and CD25, T_reg_ cell markers, in the colon and MLN was comparable between WT → *Rag1*^−/−^ and *Odc*^−/−^ → *Rag1*^−/−^ mice (Figures S2E and S2F). These data confirmed a critical role for ODC in ensuring correct T_H_ lineage fidelity both *in vitro* and *in vivo*.

### *Odc*-ΔT mice exhibit defective T_H_17 polarization in an *in vivo* model of T_H_17 induction

We further tested the role of polyamine synthesis with an *in vivo* model of T_H_17 induction in the small intestine. Anti-CD3 antibody treatment drives activation induced cell death in T cells and subsequent engulfment of apoptotic T cells by macrophages leads to IL-6 and TGF-β production, cytokines important for T_H_17 cell development (Esplugues et al., 2011). These conditions lead to small intestine inflammation and robust T_H_17 cell formation (Esplugues et al., 2011). Anti-CD3 treatment of WT mice increased small intestine CD4^+^ T cells expressing IL-17A, IL-17F, and co-expressing these cytokines (Figures 3G and S2G). However, both the frequency and number of CD4^+^ T cells expressing IL-17A and IL-17A/IL-17F was significantly lower in *Odc*-ΔT mice following anti-CD3 treatment (Figures 3G and S2G). Following anti-CD3 treatment, more than 70% of small intestine CD4^+^ T cells in WT mice expressed RORγt, but the number and frequency was lower in *Odc*-ΔT mice (Figures 3H and S2H). These data suggest a crucial role for polyamine metabolism in T_H_17 cell formation *in vivo*.

### Synthesis of the amino acid hypusine via the enzyme DHPS underlies the core requirement for polyamine metabolism in directing T_H_ lineage fidelity

**Figure 4:**
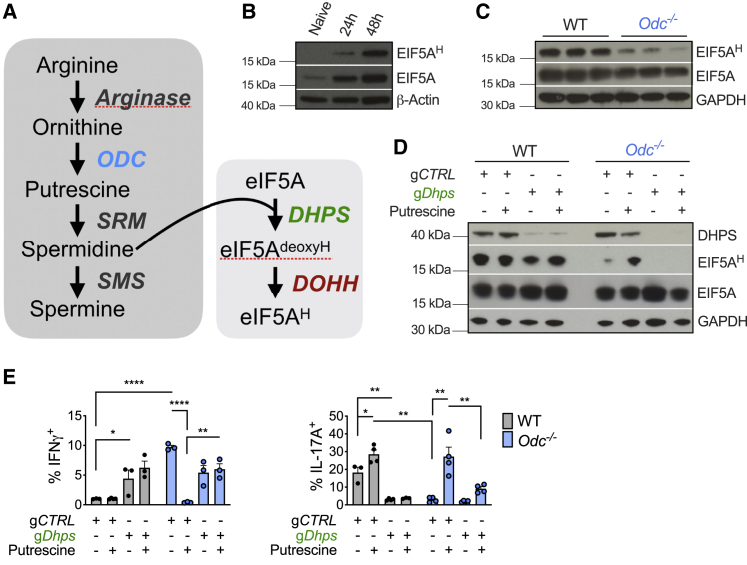
Synthesis of the amino acid hypusine underlies the central requirement for polyamine synthesis in T_H_ lineage commitment (A) PA synthesis and its role in eIF5A hypusination. (B) Immunoblot of total EIF5A and hypusinated (EIF5A^H^) in CD4^+^ T cells from WT mice activated for indicated time or rested overnight in 10 ng/mL IL-7 (naive). Representative of 3 biological replicates. (C) Immunoblots of WT and *Odc*^−/−^ naive CD4^+^ T cells activated for 48 h. Representative of 3 biological replicates. N.B., same loading control and samples were used as in Figure 2A. (D and E) Naive WT and *Odc*^−/−^ CD4^+^ T cells electroporated with g*Dhps* or gCTRL and Cas9. Cells were then activated under T_H_17 conditions ±250 μM putrescine. After 96 h, proteins were assessed by (D) immunoblot and (E) cytokines expression analyzed by FC. All data are mean ± SEM (p^∗^ < 0.05, p^∗∗^ < 0.005, p^∗∗∗^ < 0.0005, p^∗∗∗∗^ < 0.00005). (B–E) Representative of 2 experiments.

Polyamines have pleiotropic roles (Igarashi and Kashiwagi, 2000), but a key function of this pathway is synthesis of the amino acid hypusine (Chattopadhyay et al., 2008). Hypusine is present in just one protein—the translation elongation factor eIF5A (Park et al., 1981), and hypusination is critical to eIF5A’s translation factor function. eIF5A is hypusinated when a conserved lysine (K50) is converted to hypusine by deoxyhypusine synthase (DHPS) and deoxyhypusine hydroxylase (DOHH) in a process requiring spermidine (Figure 4A) (Abbruzzese et al., 1986; Park et al., 1981; Wolff et al., 1995). Here, spermidine is a substrate for DHPS, which mediates the first, rate limiting step in hypusine synthesis. We questioned whether the mechanism through which polyamines control T_H_ cell subset fidelity is via spermidine production and eIF5A hypusination. Both eIF5A and its hypusinated form (eIF5A^H^) increased after CD4^+^ T cell activation (Figure 4B). eIF5A^H^, but not total eIF5A, was decreased in *Odc*^−/−^ CD4^+^ T cells (Figure 4C), highlighting the relationship between polyamines and eIF5A^H^.

We reasoned that if hypusine was important for T_H_ differentiation, putrescine add-back would no longer rescue the *Odc*^−/−^ T cell phenotype if DHPS was absent. We generated WT and *Odc*^−/−^ T_H_17 cells in which some cells were also deleted for *Dhps* using CRISPR-Cas9. Guides targeting *Dhps* (g*Dhps*) ablated DHPS in WT and *Odc*^−/−^ T_H_17 cells compared to cells with control guides (gCTRL) (Figure 4D). *Dhps* deletion in WT T_H_17 cells reduced eIF5A^H^, but not total eIF5A levels (Figure 4D). As observed in Figure 4C, *Odc*^−/−^ T cells had reduced eIF5A^H^ with or without DHPS (Figure 4D). Importantly, putrescine restored eIF5A^H^ in *Odc*^−/−^ T cells with gCTRL, but not in *Odc*^−/−^ T cells lacking *Dhps* (Figure 4D).

*Dhps* deletion in WT T_H_17 cells increased IFN-γ (Figure 4E), phenocopying *Odc* knockout. As in Figure 2F, putrescine restored IFN-γ expression back to WT levels in *Odc*^−/−^ T_H_17 cells, but not in *Dhps*-deleted *Odc*^−/−^ T_H_17 cells (Figure 4E). Moreover, *Dhps* deletion in WT T_H_17 cells abolished IL-17A, again phenocopying *Odc*^−/−^ T_H_17 cells. Exposing *Odc*^−/−^ T_H_17 cells to putrescine restored IL-17A synthesis, an effect lost when *Dhps* was absent (Figure 4E). These data demonstrate that hypusine synthesis, via DHPS, is central to how polyamine metabolism enforces T_H_ lineage fidelity.

### *Dohh*^−/−^ CD4^+^ T_H_ cells express non-canonical cytokines and transcription factors across T_H_ cell subsets

If hypusine synthesis was important, deletion of the hypusine synthesis enzyme DOHH should phenocopy *Odc* and *Dhps* loss with aberrant T_H_ differentiation. We crossed mice containing loxP flanked exons 2–4 of *Dohh* (Sievert et al., 2014) with mice expressing *CD4*^*cre*^, to generate mice with *Dohh* deleted in T cells (*Dohh*-ΔT mice). Control mice were absent for cre recombinase. DOHH deletion in T cells decreased eIF5A^H^, but not total eIF5A (Figure S2I). *Dohh*-ΔT mice exhibited a ∼50% reduction in T cell frequency and number in spleen relative to WT littermates (Figure S2J), although naive and memory CD4^+^ T cell frequencies were comparable (Figure S2K).

**Figure 5:**
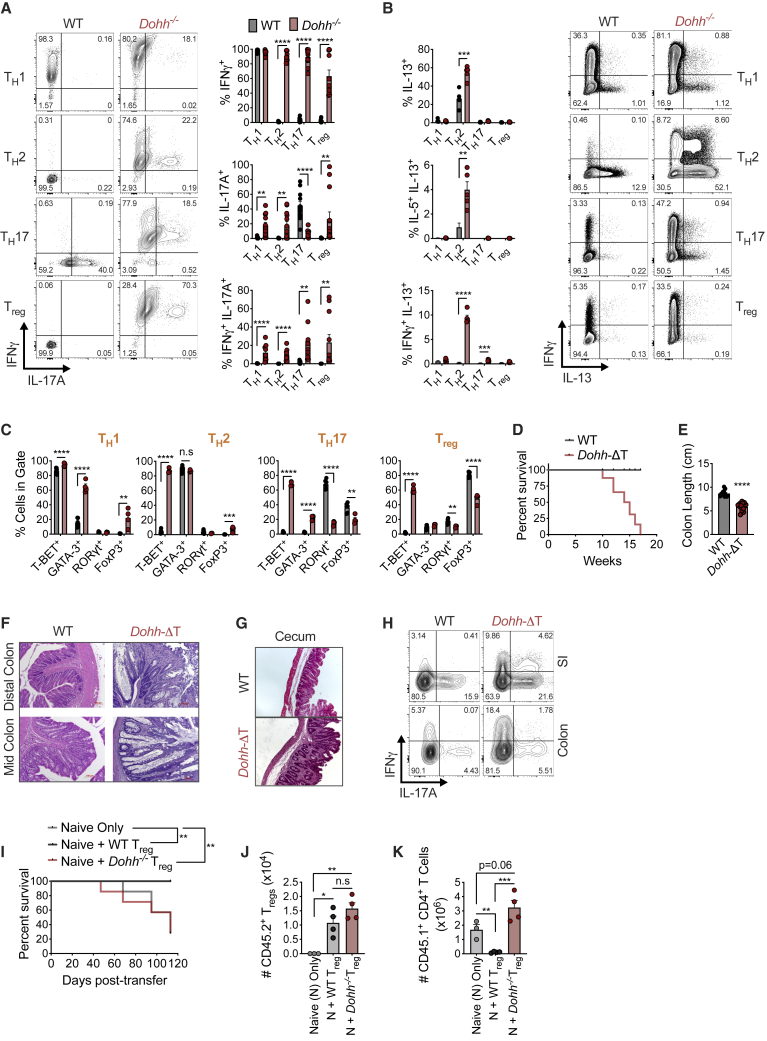
T cell-specific deletion of the hypusine-synthesizing enzyme *Dohh* leads to T cell dysregulation and colitis (A–C) Naive WT and *Dohh*^−/−^ CD4^+^ T cells polarized for 96 h and cytokine levels (A and B) and TF expression (C) analyzed by FC, representative contour plots shown. (D and E) Post-natal survival (D) and colon length (E) of WT and *Dohh*-ΔT mice. (F and G) H&E staining of mid and distal colon (F) and cecum (G) from 9-week-old WT or *Dohh*-ΔT mice. (H) CD4^+^ T cells harvested from the indicated organ of 8-week-old WT and *Dohh*-ΔT mice and cytokine expression assessed. (I) Naive CD45.1^+^CD4^+^ T cells ± WT or *Dohh*^−/−^ CD45.2^+^CD4^+^CD25^+^ T cells were sorted and transferred into *Rag1*^−/−^ mice. Clinical disease was tracked and mice sacrificed when pre-determined criteria were met (see STAR Methods). Statistics are Mantel-Cox tests. (J and K) Donor T cells were transplanted into *Rag1*^−/−^ recipient mice as in (I). Numbers of CD45.2^+^CD4^+^ T_reg_ cells (J) and CD45.1^+^CD4^+^ T cells (K) present in the colon of surviving mice on day 113 post-transfer. All data are mean ± SEM (p^∗^ < 0.05, p^∗∗^ < 0.005, p^∗∗∗^ < 0.0005, p^∗∗∗∗^ < 0.00005). (A–C) Represents 5, (H) 3, and (I) 1 experiment(s). See also Figures S2, S3, S4, and S5.

**Figure S3:**
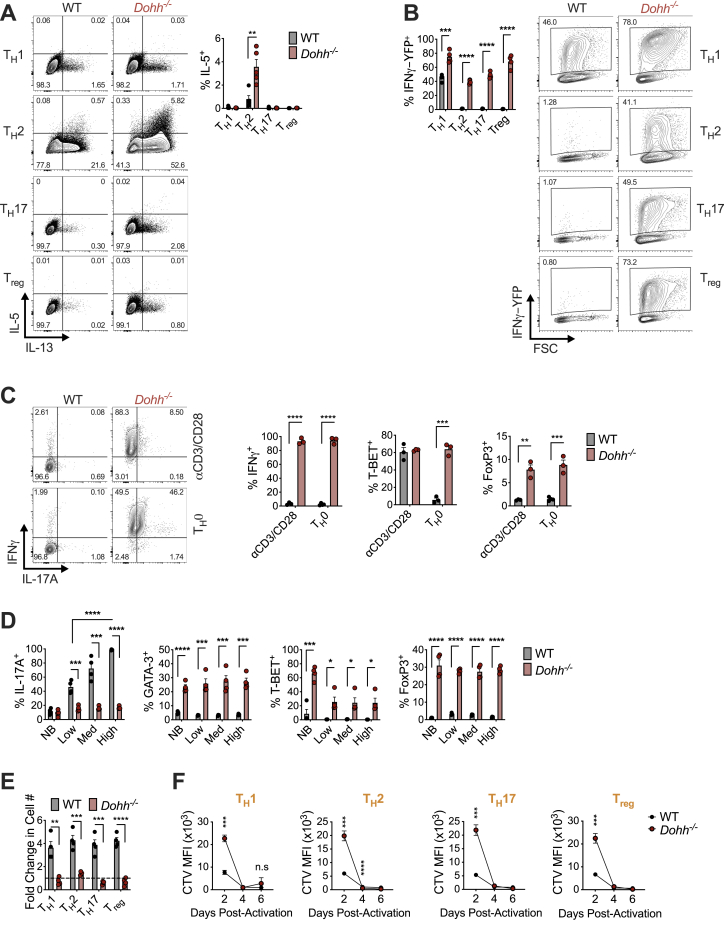
*Dohh*^*−/−*^ CD4^+^ T cells exhibit a broad pattern of dysregulated cytokine and transcription factor expression *in vitro*, related to Figure 5 (A) Naive WT and *Dohh*^−/−^ CD4^+^ T cells polarized for 96h and assayed for the expression of designated cytokine by FC after 5h of PMA/ionomycin re-stimulation. (B) Naive CD4^+^ T cells from WT and *Dohh*-ΔT-Great mice polarized for 96h and examined for YFP expression. Representative contour plots are shown. (C) Naive WT and *Dohh*^−/−^ CD4^+^ T cells activated with anti-CD3/CD28 ± anti-IFN-γ/IL-4 (T_H_0) assayed for the indicated cytokine and transcription factor 4 days post-activation. (D) Naive WT and *Dohh*^−/−^ CD4^+^ T cells polarized under T_H_17 conditions ± varying concentrations (low, medium, high, or no blocking - see methods) of blocking antibodies. After 96h, expression of the indicated cytokine or transcription factors was assessed. (E) Fold change in the number of WT and *Dohh*^*−/−*^ T_H_ cells between day 0 and day 4 of *in vitro* culture. (F) Naive WT and *Dohh*^*−/−*^ CD4^+^ T cells were stained with cell trace violet (CTV) proliferation dye, polarized into T_H_ subsets, and assessed for CTV levels by FC on the indicated day. All data are mean ± SEM (p^∗^ < 0.05, p^∗∗^ < 0.005, p^∗∗∗^ < 0.0005, p^∗∗∗∗^ < 0.00005. (A, D) Representative of 3 experiments, (D-G, I-N) (B,C, E,F) Representative of 2 experiments.

We sorted naive CD4^+^ T cells from *Dohh*-ΔT mice and activated and polarized them *in vitro* into T_H_1, T_H_2, T_H_17, and T_reg_ cells. IFN-γ was remarkably elevated in *Dohh*^−/−^ T_H_2, T_H_17, and T_reg_ cells (Figure 5A). There was also an increased frequency of cells that co-produced IFN-γ and IL-17A in all T_H_ cell subsets (Figure 5A) and enhanced secretion of IFN-γ and IL-17A together with other cytokines (TNF, IL-22, and IL-6) in *Dohh*^−/−^ T cells (Figures S2L and S2M). We also observed an increased frequency of cells expressing IL-5 and/or IL-13 in *Dohh*^−/−^ T cells under T_H_2 conditions (Figures 5B and S3A), including a population of cells co-producing IFN-γ and IL-13 in T_H_2 and T_H_17 conditions (Figure 5B). Similar to *Odc*^−/−^ T cells (Figure 2C), IL-17A expression was ablated in *Dohh*^−/−^ T_H_17 cells (Figure 5A).

To test if *Dohh*^−/−^ T_H_ cells had dysregulated cytokine expression without restimulation, we crossed *Dohh*-ΔT to Great mice containing an IRES-enhanced YFP construct after the IFN-γ stop codon (Reinhardt et al., 2009). This allows expression of both IFN-γ and eYFP from the same mRNA and analysis of IFN-γ-expressing cells without restimulation. We polarized naive CD4^+^ T cells from *Dohh*-ΔT-Great mice into various T_H_ cells and assessed eYFP expression after 4 days. Naive CD4^+^ T cells from littermates that expressed Great alleles, but lacked cre recombinase, were controls. All T_H_ subsets displayed enhanced IFN-γ in the absence of DOHH (Figure S3B), suggesting that the dysregulated cytokine response in *Dohh*^−/−^ T_H_ cells is apparent prior to, and in the absence of, restimulation.

**Figure S4:**
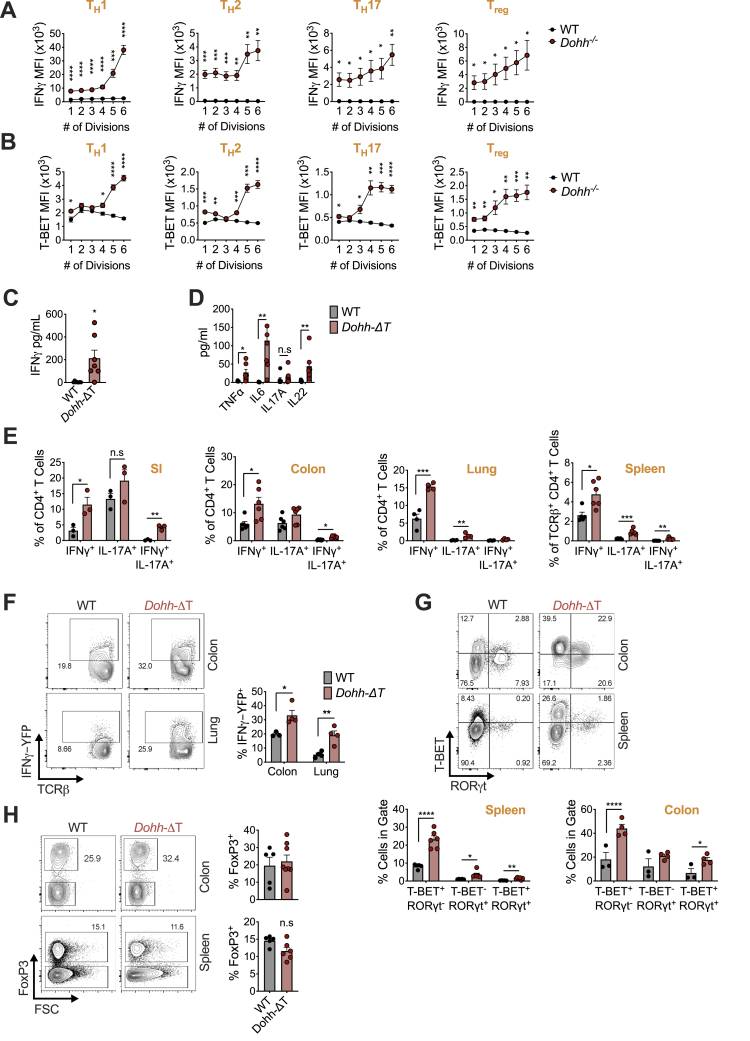
T cells from *Dohh*-ΔT mice exhibit perturbed phenotypes across multiple organs, related to Figure 5 Naive WT and *Dohh*^*−/−*^ CD4^+^ T cells stained with CTV, polarized, and then assessed for (A) IFN-γ after restimulation or (B) T-Bet expression on day 4 of culture. (C,D) Serum from 6-10 week old WT and *Dohh*-ΔT mice assayed by cytokine bead array. (E) CD4^+^ T cells harvested from designated organs of WT and *Dohh*-ΔT and cytokine expression analyzed by FC following 4h re-stim (SI = small intestine). (F) CD4^+^ T cells harvested from designated organs of 7 week old *Dohh*-ΔT-Great mice and their WT littermate controls and YFP expression analyzed. Representative dot plots are shown and gated on CD45^+^ TCRβ^+^ cells. (G,H) CD4^+^ T cells harvested from designated organs of WT and *Dohh*-ΔT mice and expression of indicated transcription factor analyzed by FC. Representative dot plots in (G) gated on Foxp3^-^ CD4^+^ T cells. All data are mean ± SEM (p^∗^ < 0.05, p^∗∗^ < 0.005, p^∗∗∗^ < 0.0005, p^∗∗∗∗^ < 0.00005). (A, B, F) is representative of two experiments, (C-E, G,H) representative of 3 experiments.

We also found increased T-bet in *Dohh*^−/−^ T cells under all polarizing conditions and an increased frequency of GATA3 expressing cells in T_H_1 and T_H_17 conditions (Figure 5C). There was dysregulated Foxp3 in all T_H_ subsets and a reduced frequency of RORγt-expressing cells under T_H_17 conditions (Figure 5C). Cytokine and TF expression was also aberrant when cells were activated solely with anti-CD3/CD28 and IL-2 or also exposed to IFN-γ and IL-4 blocking antibodies (T_H_0) (Figure S3C). Even under increasing concentrations of blocking antibodies targeting numerous cytokines, many lineage-specific TFs were still perturbed in *Dohh*^−/−^ T_H_17 cells and IL-17A expression could not be restored to WT levels (Figure S3D). Like *Odc*^−/−^ T_H_ cells, *Dohh* loss delayed T cell expansion (Figures S3E and S3F), and IFN-γ and T-bet levels were altered across all *Dohh*^−/−^ T_H_ cells subsets regardless of cell division (Figures S4A and S4B).

The robust phenotypic overlap between *Odc*^−/−^ and *Dohh*^−/−^ T cells strongly supports that the requirement for polyamine metabolism in enforcing T_H_ cell lineage fidelity is due to spermidine acting as a substrate for hypusine synthesis.

### Mice with a T cell-specific deletion of *Dohh* exhibit T cell dysregulation, inflammation, and colitis

Remarkably, *Dohh*-ΔT mice died at ∼10–17 weeks of age (Figure 5D). IFN-γ and other cytokines were significantly increased in the serum of *Dohh*-ΔT mice (Figures S4C and S4D), correlating with disease. Colon length was decreased in *Dohh*-ΔT mice (Figure 5E), accompanied by increased immune infiltrates, altered villi structure (Figure 5F), and cecal thickening (Figure 5G), indicative of colitis, a T cell-driven pathology (Powrie et al., 1994). T cells isolated from various organs of *Dohh*-ΔT mice displayed increased frequencies of CD4^+^ T cells producing IFN-γ, and IFN-γ and/or IL-17 (Figures 5H and S4E), directly correlating intestinal inflammation with increased cytokine-producing T cells *in vivo* (Harbour et al., 2015; Park et al., 2005; Wang et al., 2015; Zielinski et al., 2012). T cells isolated from colon and lung of *Dohh*-ΔT-Great mice had enhanced IFN-γ relative to WT controls even without restimulation (Figure S4F). Notably, *Dohh*-ΔT mice had an increased frequency of CD4^+^ T cells expressing T-bet and/or RORγt in the colon or spleen (Figure S4G), without any difference in the frequency of Foxp3-expressing T cells (Figure S4H).

### T cell-specific deletion of *Dhps* leads to T cell dysregulation, inflammation, and colitis

**Figure S5:**
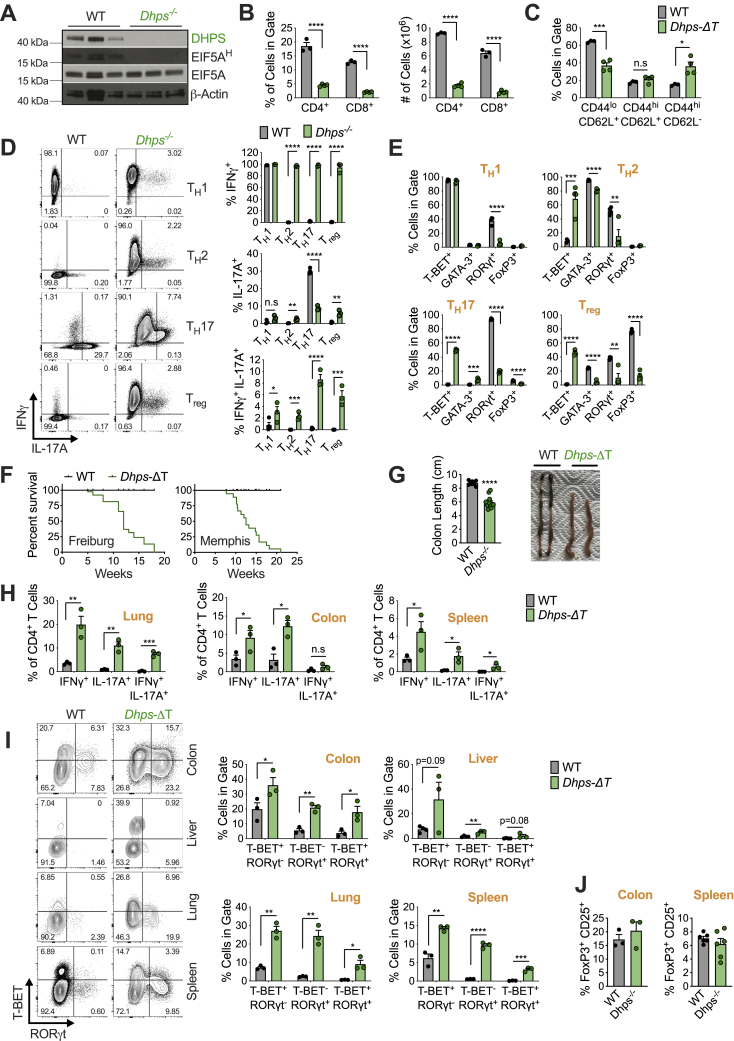
T cell-specific deletion of *Dhps* confirms a role for hypusine synthesis in T_H_ lineage fidelity, related to Figure 5 (A) Immunoblot of WT and *Dhps*^*−/−*^ CD4^+^ T cells activated for 48h with anti-CD3/CD28. (B) Frequency/absolute numbers of T cells and (C) frequency of naive/memory CD4^+^ T cells in the spleen of 7 week old *Dhps*-ΔT mice and their WT littermates. (D) WT and *Dhps*^−/−^ naive CD4^+^ T cells polarized for 96h. Cytokine expression was assessed by FC 5 hours after re-stimulation, representative contour plots are shown. (E) WT and *Dhps*^−/−^ naive CD4^+^ T cells polarized as in (D) and labeled transcription factor assessed by FC. (F) Post-natal survival of WT and *Dhps*-ΔT mice from Freiburg or Memphis facilities. (G) Colon length and representative images from 6 week old WT and *Dhps*-ΔT mice. (H) CD4^+^ T cells harvested from the designated organ of WT and *Dhps*-ΔT and cytokine expression analyzed by FC. (I,J) CD4^+^ T cells harvested from indicated organ of WT and *Dhps*-ΔT mice and the frequency of CD4^+^ T cells expressing labeled transcription factor assessed. Analysis is gated on Foxp3^-^ CD4^+^ T cells. Representative contour plots are shown. (K) Frequency of T_regs_ among CD4^+^ T cells in colon and spleen of WT and *Dhps*-ΔT. All data are mean ± SEM (p^∗^ < 0.05, p^∗∗^ < 0.005, p^∗∗∗^ < 0.0005, p^∗∗∗∗^ < 0.00005). (A) Representative of 1 experiment, (B-F) Representative of 3 experiments, (H-J) Representative of 4 experiments.

The similar *Odc*^−/−^ and *Dohh*^−/−^ T cell phenotypes indicated that polyamine synthesis in T_H_ cell subset specification was mechanistically linked to hypusine synthesis. We reasoned that we should therefore observe a similar phenotype to *Odc*^−/−^ and *Dohh*^−/−^ T cells in a third genetic model - *Dhps* deficiency (Figure 4A). We crossed mice containing loxP flanked exons 2-7 of the *Dhps* gene (Pällmann et al., 2015) with mice expressing *CD4*^*cre*^, to generate mice deleted for *Dhps* in T cells (*Dhps*-ΔT mice). Control mice were absent for cre recombinase. Deletion of DHPS in T cells decreased eIF5A^H^ but not total eIF5A (Figure S5A). Like *Dohh*-ΔT mice, *Dhps*-ΔT mice had reduced T cell frequency and numbers in spleen (Figure S5B), although had they enhanced effector memory CD4^+^ T cell frequency (Figure S5C). Like *Odc*^−/−^ and *Dohh*^−/−^ T cells, *in vitro* activated *Dhps*-ΔT CD4^+^ T cells exhibited markedly elevated IFN-γ, with increased frequencies of cells producing both IFN-γ and IL-17A while IL-17A synthesis was defective in the T_H_17 lineage (Figure S5D). Lineage-defining TF expression was dysregulated across all T_H_ subsets (Figure S5E). As with *Dohh*-ΔT mice, two independently generated *Dhps*-ΔT mouse lines in distinct animal facilities developed fatal disease at around 10–20 weeks of age (Figure S5F) with intestinal inflammation as measured by decreased colon length (Figure S5G). *Dhps*-ΔT mice also displayed increased frequencies of IFN-γ, and IFN-γ and/or IL-17 producing CD4^+^ T cells in the lung, colon, and spleen (Figure S5H), with increased T-bet and/or RORγt expression in multiple tissues (Figures S5I and S5J), correlating with their inflammatory phenotype (Harbour et al., 2015; Neurath et al., 2002; Yen et al., 2006), yet no change in Foxp3-expressing cells (Figure S5K).

To test if failed suppression of aberrant T cell activity by T_regs_ contributed to the fatal inflammation in *Dohh*-ΔT and *Dhps*-ΔT mice, we transferred naive WT CD45.1^+^ CD4^+^ T cells into *Rag1*^−/−^ recipients to initiate colitis, together with T_regs_ sorted from WT and *Dohh*-ΔT mice. In this model, co-transfer of T_regs_ protects against disease (Mottet et al., 2003). Transfer of naive CD4^+^ T cells alone efficiently induced colitis in recipient mice, limiting survival (Figure 5I). Co-transfer of WT T_regs_ shielded recipient mice from disease (Figure 5I), but *Dohh*^*−*/−^ T_reg_ transfer had no such effect, and recipient mice died at the same rate as mice that received no T_regs_ (Figure 5I). We found similar numbers of WT and *Dohh*^−/−^ T_regs_ in surviving recipient mice 113 days post-transfer (Figure 5J), suggesting lost suppressive function in *Dohh*^−/−^ T_regs_ rather than impaired proliferation or survival of these cells. This was highlighted by the ability of WT, but not *Dohh*^−/−^, T_regs_ to suppress naive CD45.1^+^ T cell expansion in recipient mice (Figure 5K). Therefore, the T cell-driven pathology in *Dohh*-ΔT and *Dhps*-ΔT mice likely reflects a cell-intrinsic failure of CD4^+^ T cells to faithfully regulate effector differentiation, coupled to a loss of T_reg_ tolerogenic competence. Collectively, these data suggest that hypusine synthesis is a critical factor controlling T_H_ differentiation and function both *in vitro* and *in vivo* where loss of this factor drives aberrant T cell responses and systemic inflammation.

### Polyamine or hypusine-deficient CD4^+^ T cells exhibit widely altered chromatin accessibility linked to broad changes in histone modifications

**Figure 6:**
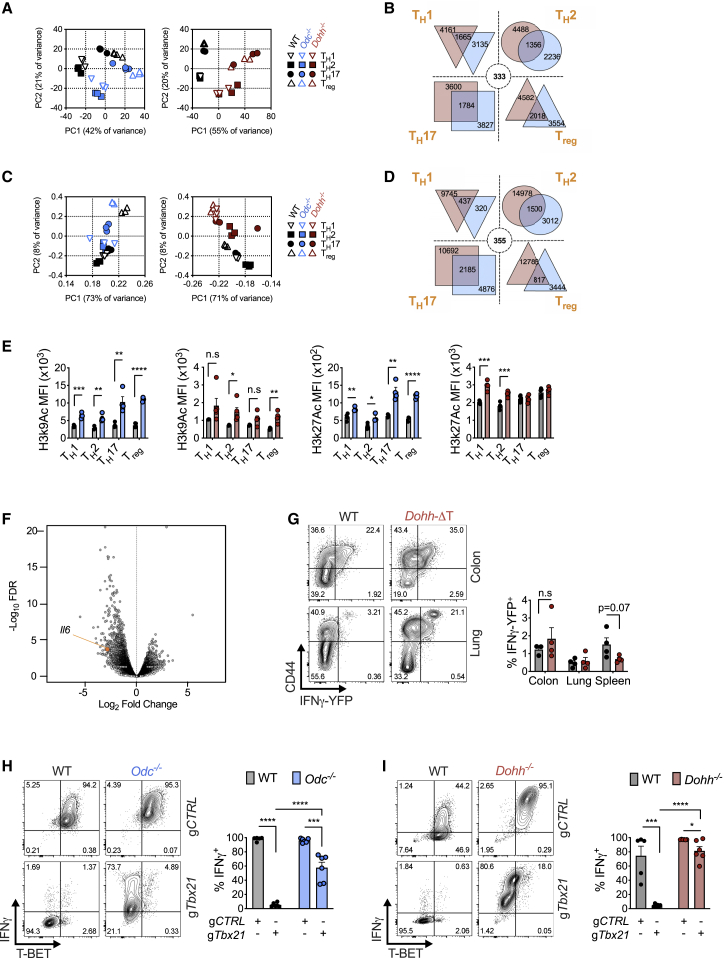
The polyamine-hypusine axis regulates the T cell epigenome to enforce appropriate T_H_ cell differentiation and function (A) RNA-seq of naive WT, *Odc*^−/−^, and *Dohh*^−/−^ CD4^+^ T cells polarized for 96 h. Effect of *Odc* and *Dohh* deficiency is shown via principal component analysis, indicating the percentage of variance allocated to each component in parenthesis. (B) Venn diagram depicting number of differentially regulated genes disparate, or shared, between *Odc*^−/−^ and *Dohh*^−/−^ CD4^+^ T cells of indicated T_H_ lineage. Dashed circle indicates number of differentially expressed genes common to all T_H_ cell subsets. (C) ATAC-seq on CD4^+^ T cells treated as in (A). (D) Venn diagram depicting number of differentially regulated regions of open chromatin disparate or shared between *Odc*^−/−^ and *Dohh*^−/−^ CD4^+^ T cells. Dashed circle indicates number of differentially regulated regions of open chromatin common to all T_H_ cell subsets. (E) WT, *Odc*^−/−^, or *Dohh*^−/−^ CD4^+^ T cells assayed for chromatin modifications by FC on day 4. Analysis performed on Ki-67^+^, diploid cells with “single” DNA content based on FxCycle (DAPI) staining in live cell gate. (F) ATAC-seq on naive CD44^lo^CD62L^+^ CD4^+^ T cells from spleens of WT and *Dohh*-ΔT mice. Volcano plots depict all differentially regulated regions of open chromatin with immunologically relevant loci in orange. (G) YFP expression assessed in CD4^+^ T cells from 7-week-old WT and *Dohh*-ΔT-Great mice. Bar graphs depict % of cells YFP^+^ in CD44^lo^ CD4^+^ T cells, representative contour plots are shown. (H and I) Naive WT and *Odc*^−/−^ CD4^+^ T cells (H) and naive WT and *Dohh*^−/−^ CD4^+^ T cells (I) electroporated with g*Tbx21* or gCTRL with Cas9 and activated under T_H_1 conditions. IFN-γ assessed by FC on day 4. All data are mean ± SEM (p^∗^ < 0.05, p^∗∗^ < 0.005, p^∗∗∗^ < 0.0005, p^∗∗∗∗^ < 0.00005). Representative of 1–3 (E), and of 2 (G–I) experiments. See also Figures S6 and S7.

**Figure S6:**
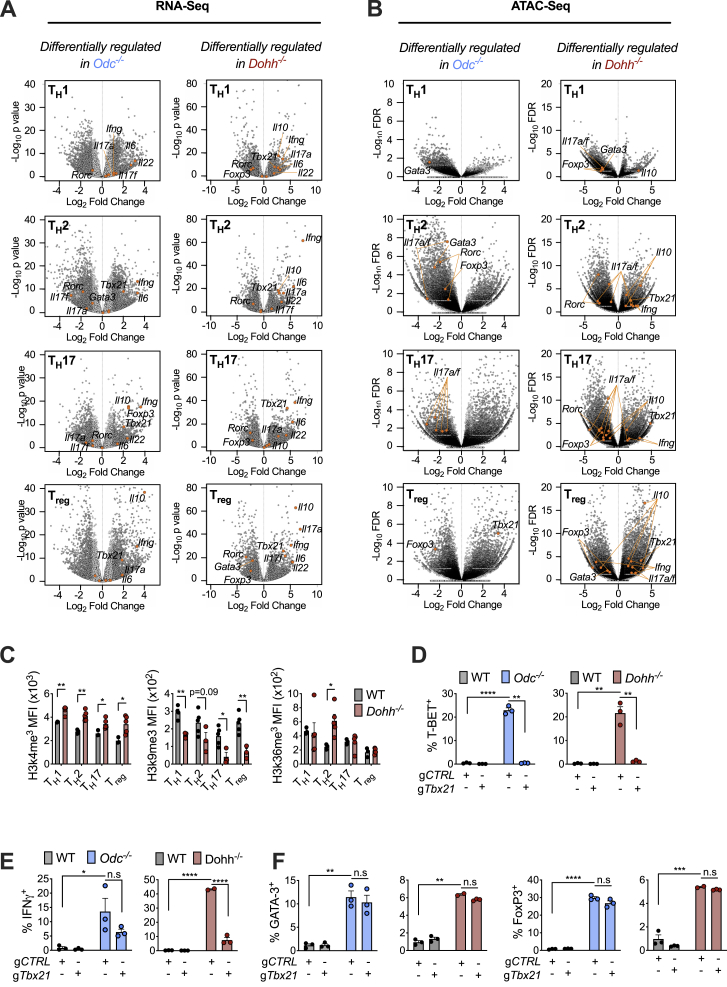
RNA-seq and ATAC-seq data from *in vitro* activated WT, *Odc*^*−/−*^, and *Dohh*^*−/−*^ CD4^+^ T_H_ cell subsets, related to Figure 6 Naive CD4^+^ T cells from WT, *Odc*-ΔT, and *Dohh*-ΔT mice polarized in indicated T_H_ conditions for 96h were assessed by (A) RNA-seq or (B) ATAC-Seq. Volcano plots depict differentially regulated genes (A) or differentially regulated regions of open chromatin (B). (C) WT and *Dohh*^*−/−*^ CD4^+^ T cells assayed for chromatin modifications by FC in indicated T_H_ cell subset 96h post-activation/polarization. Analysis was performed on Ki-67^+^ cells and diploid cells with ‘single’ DNA content based on FxCycle (dapi) staining in the live cell gate. (D-F) Naive WT, *Odc*^*−/−*^ and *Dohh*^*−/−*^ CD4^+^ T cells electroporated with guide RNAs specific for *Tbx21* (g*Tbx21*) or control guide (gCTRL) with Cas9 nuclease and activated under T_H_17 conditions. After 4 days, levels of indicated cytokine or transcription factor assessed by FC. All data are mean ± SEM (p^∗^ < 0.05, p^∗∗^ < 0.005, p^∗∗∗^ < 0.0005, p^∗∗∗∗^ < 0.00005). (C) is representative of 1-3 experiments, (D-F) is representative of 2 experiments.

In addition to selective TF and cytokine expression, a key feature of T_H_ cell differentiation is the ability of one program to silence other T_H_ effector fates (e.g., T_H_1 cells not only produce IFN-γ but repress IL-4 and IL-17 synthesis). Various transcriptomic and epigenomic mechanisms underlie this lineage commitment fidelity (Kanno et al., 2012). Because *Odc-*, *Dohh-*, and *Dhps*-deficient T cells displayed attributes of multiple T_H_ lineages simultaneously, we reasoned these cells likely had wide-scale transcriptomic/epigenomic changes. We examined gene transcription by RNA sequencing (RNA-seq), finding a dramatically altered transcriptional profile in both *Odc*^−/−^ and *Dohh*^−/−^ cells relative to WT cells across all T_H_ cell subsets (Figure 6A) with many genes commonly differentially regulated in both genotypes (Figure 6B) including essential differentiation and effector function genes (Figure S6A). Epigenetic status is an important regulator of gene expression and cellular differentiation (Allis and Jenuwein, 2016). To investigate if chromatin changes drove transcriptional perturbations in *Odc*^−/−^ and *Dohh*^−/−^ T_H_ cells, we performed ATAC-seq across genotypes and T_H_ cell subsets. Chromatin accessibility, which influences the access of T_H_ cell subset-specific TFs driving expression of lineage-specific genes (Hirahara et al., 2011), was highly disparate between all *Odc*^−/−^ and *Dohh*^−/−^ T_H_ cell subsets compared to WT cells (Figure 6C). Across T_H_ cell subsets, many differentially regulated chromatin regions were shared between *Odc*^−/−^ and *Dohh*^−/−^ T_H_ cells (Figure 6D), including loci critical for T_H_ cell differentiation and lineage identity, such as *Tbx21* (T-Bet), *Gata3*, *Rorc*, *Ifng*, and *Il17* (Figure S6B). These data show that *Odc*^−/−^ and *Dohh*^−/−^ T cells exhibit substantially remodeled chromatin and indicate a prominent role for the polyamine-hypusine axis in governing the T cell epigenome.

To examine what initiated altered chromatin accessibility, we measured epigenetic marks on histone proteins, including activating modifications such as H3k4me^3^, H3k9Ac, H3k27Ac, and H3k36me^3^, and the repressive mark H3k9me^3^ (Bannister and Kouzarides, 2011; Lawrence et al., 2016). Many histone marks were dysregulated in all *Odc*^−/−^ and *Dohh*^−/−^ T_H_ cell subsets, compared to WT cells (Figures 6E and S6C).

Next, we sought to address if this altered chromatin was apparent in naive T cells or was coupled to T cell activation. ATAC-seq of splenic naive T cells of *Dohh*-ΔT mice and their WT littermates revealed a level of chromatin remodeling already present in naive *Dohh*^−/−^ T cells, although we did not observe changes in chromatin accessibility in any T_H_ lineage commitment-relevant loci, with the exception of *Il6* (Figure 6F). Remodeling in naive *Dohh*^−/−^ T cells (2,388 differentially regulated regions) was comparatively minor compared to that of differentiated *Dohh*^−/−^ T_H_ subsets (9,745–14,978 differentially regulated regions). To confirm that expression of factors associated with T_H_ differentiation was not already differentially regulated in naive *Dohh*^−/−^ T cells, we used *Dohh*-ΔT-Great mice to assess IFN-γ in naive and effector CD4^+^ T cells. IFN-γ-YFP expression was restricted solely to CD44-expressing effector CD4^+^ T cells in the colon, spleen, and lungs of both *Dohh*-ΔT-great mice and WT littermate controls (Figure 6G). These data suggest that although chromatin dynamics are altered in *Dohh*^−/−^ naive T cells, the major remodeling during perturbed T_H_ differentiation is coupled to T cell activation.

### Preventing aberrant T-bet expression does not restore focused T_H_ lineage commitment in *Odc*^−/−^ and *Dohh*^−/−^ CD4^+^ T cells and supports a key role for polyamine metabolism in governing the T cell epigenome

**Figure S7:**
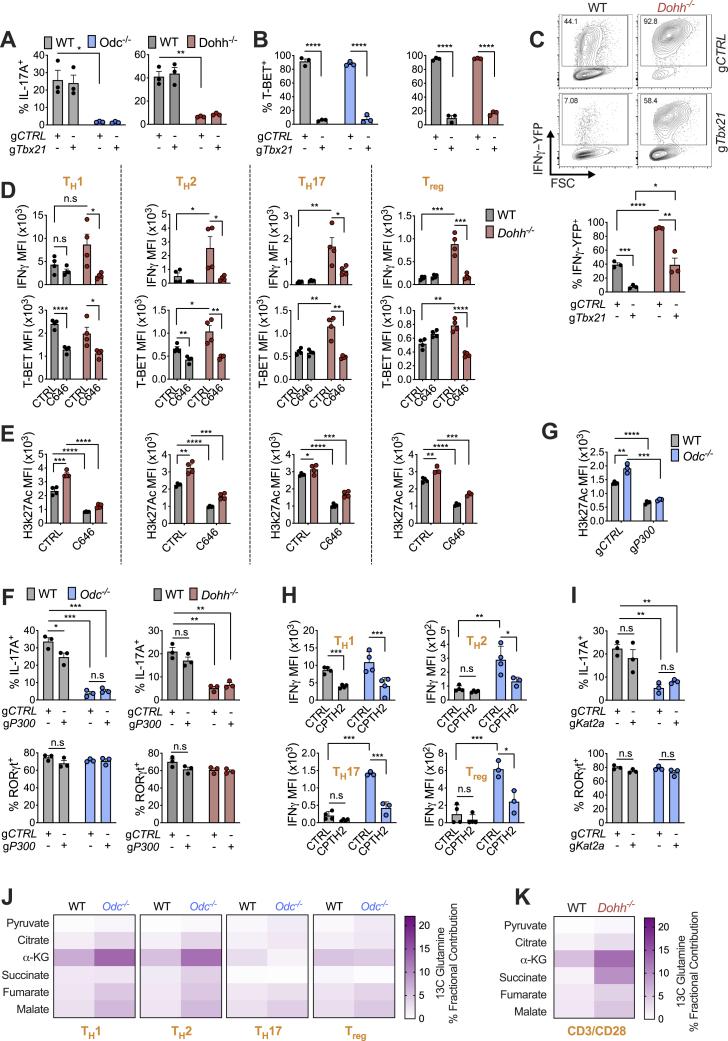
HAT inhibition or deletion restores efficient T_H_ differentiation in *Odc*^*−/−*^ and *Dohh*^*−/−*^ CD4^+^ T cells, related to Figures 6 and 7 (A,B) Naive WT, *Odc*^*−/−*^ and *Dohh*^*−/−*^ CD4^+^ T cells deleted for *Tbx21* using CRISPR Cas9 and polarized in T_H_1 conditions. Levels of labeled protein was assessed by FC after 96h. (C) Naive CD4^+^ T cells from WT and *Dohh*-ΔT-Great mice deleted for *Tbx21* as described in (A) and polarized for 96h in T_H_1 conditions. YFP expression analyzed by FC, representative contour plots are shown. (D, E) Naive WT and *Dohh*^*−/−*^ CD4^+^ T cells polarized in indicated condition for 72 hours. Cells were treated with 20 μM C646 for the final 48h of culture and assayed for (D) IFN-γ and T-bet or (E) H3k27 acetylation levels by FC. Analysis in (E) performed on Ki-67^+^ cells and diploid cells with ‘single’ DNA content based on FxCycle (dapi) staining in live cell gate. (F) Naive WT, *Odc*^*−/−*^ and *Dohh*^*−/−*^ CD4^+^ T cells electroporated with g*P300* or a gCTRL with Cas9 nuclease and activated under T_H_17 conditions. Protein expression was assessed after 96h. (G) Naive WT and *Odc*^*−/−*^ CD4^+^ T cells were treated as in (F), after 96h H3k27 acetylation assessed by FC. (H) Naive WT and *Odc*^*−/−*^ CD4^+^ T cells polarized for 72. Cells were treated with 20 μM CPTH2 for final 48h of culture and assayed for IFN-γ after 5h restimulation. (I) Naive WT and *Odc*^*−/−*^ CD4^+^ T cells electroporated with g*Kat2a* or gCTRL with Cas9 nuclease and activated under T_H_17 conditions. Indicated protein was assessed on day 4. (J) Naive WT and *Odc*^*−/−*^ CD4^+^ T cells cultured for 72h under different polarizing conditions then exposed to 4 mM ^13^C glutamine for 24h. Tracing of glutamine into stated metabolites was performed by mass spectrometry. (K) Naive WT and *Dohh*^*−/−*^ CD4^+^ T cells activated with anti-CD3/CD28 for 72h then treated as in (J) for the flux of glutamine carbons into TCA cycle metabolites. All data are mean ± SEM (p^∗^ < 0.05, p^∗∗^ < 0.005, p^∗∗∗^ < 0.0005, p^∗∗∗∗^ < 0.00005). (A-C, F-I) Representative of two experiments, (D) Representative of 3 experiments, (E) is representative of one experiment.

T-bet can regulate T_H_1 differentiation by recruiting chromatin modifying enzymes promoting permissive chromatin marks at loci required for the T_H_1 program (Lazarevic et al., 2013). Both *Odc*^−/−^ and *Dohh*^−/−^ T_H_ cells have increased T-bet, which could promote chromatin modifying enzyme recruitment. We polarized *Odc*^−/−^ and *Dohh*^−/−^ T cells under T_H_17 conditions and used CRISPR-Cas9 to ablate *Tbx21* (encoding T-bet). Although *Tbx21*-deletion was efficient (Figure S6D) and reduced IFN-γ expression to WT levels (Figure S6E), T-bet-deficient *Odc*^−/−^ and *Dohh*^−/−^ T cells still lacked T_H_17 lineage commitment, highlighted by increased GATA3 and Foxp3 (Figure S6F). Moreover, maintaining IL-17 synthesis requires inhibition of T-bet expression (Kanno et al., 2012; Lazarevic et al., 2013), but reducing aberrant T-bet levels in *Odc*^−/−^ and *Dohh*^−/−^ T_H_17 cells failed to restore IL-17A (Figure S7A).

We also assessed T_H_1 differentiation in *Odc*^−/−^ and *Dohh*^−/−^ T cells lacking T-bet (Figure S7B). *Tbx21* deletion abrogated IFN-γ expression in WT T_H_1 cells, but had remarkably little impact on IFN-γ synthesis in *Odc*^−/−^ and *Dohh*^−/−^ T_H_1 cells (Figures 6H and 6I), a phenomenon also seen when we deleted *Tbx21* in naive T cells isolated from *Dohh*-ΔT-Great mice and polarized under T_H_1 conditions. Although *Tbx21*-deletion nullified IFN-γ-YFP expression in WT T_H_1 cells, T-bet-deficient *Dohh*^−/−^ T_H_1 cells still expressed significant IFN-γ-YFP, even without restimulation (Figure S7C). These data suggest that in some contexts, T-bet is not an absolute requirement for CD4^+^ T cell IFN-γ production. The capacity of *Odc*^−/−^ and *Dohh*^−/−^ T_H_1 cells to synthesize IFN-γ in the absence of T-bet likely reflects remodeling at the *Ifng* locus such that T-bet binding is no longer mandatory for IFN-γ transcription, supporting the notion that polyamine metabolism, via hypusine synthesis, is a central regulator of the T cell epigenome. This observation also suggests that the dramatic dysregulation of T-bet expression in polyamine- and hypusine-deficient T_H_ cells does not explain aberrant T_H_ differentiation.

### Reducing histone acetylation restores faithful T_H_ lineage commitment in polyamine and hypusine-deficient CD4^+^ T cells

**Figure 7:**
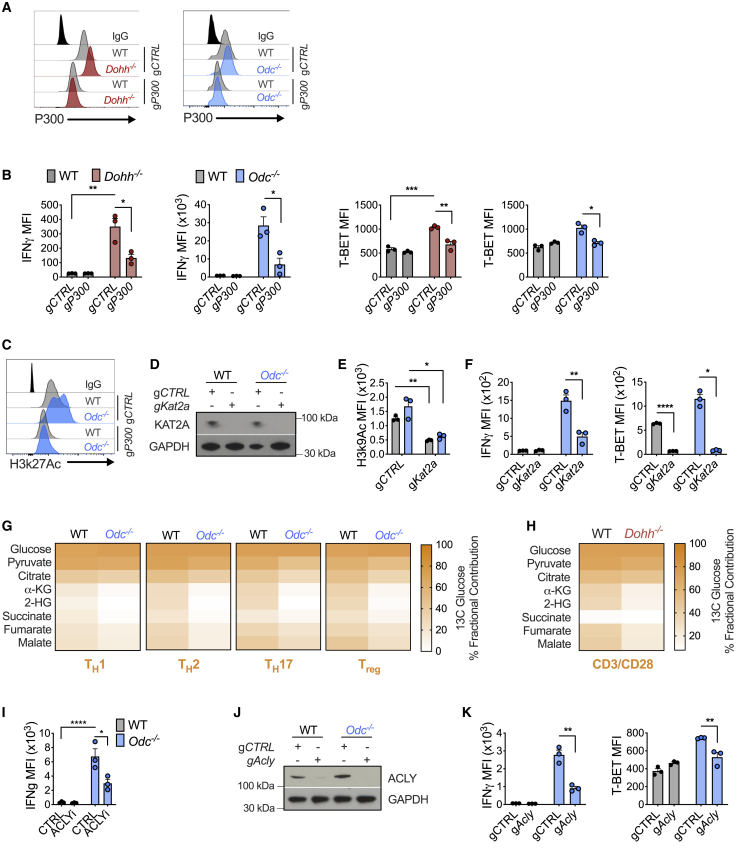
Histone acetyltransferases and a rewired TCA cycle govern the remodeled epigenome in *Odc*^−/−^ and *Dohh*^−/−^ CD4^+^ T cells (A) Naive CD4^+^ T cells from *Odc*-ΔT and *Dohh*-ΔT mice and their littermate controls were electroporated with g*P300* or gCTRL with Cas9 and activated under T_H_17 conditions for 96 h. Representative histograms of P300 levels are shown. (B and C) T-bet and IFN-γ expression (B) and H3k27 acetylation levels (C) in WT, *Odc*^−/−^, or *Dohh*^−/−^ T cells as treated in (A). (D–F) Naive WT and *Odc*^−/−^ CD4^+^ T cells electroporated with g*Kat2a* or gCTRL with Cas9 and activated under T_H_17 conditions. After 96 h, proteins were assessed by immunoblot (D) or FC (E and F). (G) Naive WT and *Odc*^−/−^ CD4^+^ T cells were polarized and after 72 h re-plated in 11 mM ^13^C glucose for 24 h. Glucose tracing performed by MS. (H) MS analysis of ^13^C glucose tracing in WT and *Dohh*^−/−^ T_H_ cells treated as in (G). (I) Naive WT and *Odc*^−/−^ CD4^+^ T cells polarized in T_H_17 conditions for 72 h. Cells were treated with 30 μM BMS303141 for the final 48 h of culture. (J) Naive WT or *Odc*^−/−^ CD4^+^ T cells electroporated with g*Acly* or gCTRL with Cas9 and activated under T_H_17 conditions. After 96 h, levels of the indicated protein were assessed by immunoblot. (K) Naive WT or *Odc*^−/−^ CD4^+^ T cells treated as in (J). After 96 h, T-bet and IFN-γ were assessed by FC. All data are mean ± SEM (p^∗^ < 0.05, p^∗∗^ < 0.005, p^∗∗∗^ < 0.0005, p^∗∗∗∗^ < 0.00005). Representative of 2 (A–F, I, and J), or 1 experiment (G and H). See also Figure S7.

We reasoned that if chromatin dysregulation in *Odc*^−/−^ and *Dohh*^−/−^ T cells was driven by observed changes in histone marks (Figure 6E), reducing histone acetylation should restore canonical expression of cytokines and TFs. We exposed *Dohh*^−/−^ T cells to C646, an inhibitor of the histone acetyltransferase (HAT) P300 that mediates H3k27 acetylation, a dysregulated mark in *Dohh*^−/−^ T cells. Treating *Dohh*^−/−^ T cells with C646 restored IFN-γ and T-bet levels across T_H_ subsets to WT levels (Figure S7D) and reduced H3k27 acetylation (Figure S7E). We then examined if ablating *P300* could also restore correct T_H_ differentiation in T cells deficient for polyamine or hypusine synthesis. T cells with guides specific for *P300* (*gP300*) displayed reduced P300 protein compared to T cells with gCTRL (Figure 7A). *P300* deletion reduced IFN-γ and T-bet in both *Odc*^−/−^ and *Dohh*^−/−^ T_H_17 cells (Figure 7B) but did not restore IL-17A (Figure S7F). Notably, reducing P300 diminished H3k27 acetylation in WT and *Odc*^−/−^ T_H_17 cells (Figures 7C and S7G).

H3k9 acetylation was also dysregulated in *Odc*^−/−^ and *Dohh*^−/−^ T_H_ cells (Figure 6E). To probe if reducing H3k9 acetylation could restore normal T_H_ lineage commitment, we inhibited the HAT KAT2A, which mediates H3k9 acetylation in T cells (Castillo et al., 2019; Gao et al., 2017; Ghosh et al., 2016; Goswami and Kaplan, 2012). The KAT2A inhibitor CPTH2 reduced IFN-γ in *Odc*^−/−^ T cells in all subsets (Figure S7H). KAT2A deletion in WT and *Odc*^−/−^ T_H_17 cells (Figure 7D) reduced H3k9 acetylation (Figure 7E) and abolished dysregulated IFN-γ and T-bet expression in *Odc*^−/−^ T_H_17 cells (Figure 7F), but did not recover IL-17A or RORγt (Figure S7I). These data imply a key role for aberrant histone acetylation, driven by HATs, in the chromatin remodeling that occurs in the absence of polyamine metabolism and/or hypusine synthesis.

### *Odc*^−/−^ and *Dohh*^−/−^ T cells reveal a dysregulated TCA cycle that may drive aberrant histone acetylation and associated chromatin remodeling

Our data suggested that aberrant histone acetylation drove the chromatin alterations precluding *Odc*^−/−^ and *Dohh*^−/−^ T cells from competent T_H_ lineage commitment. The tricarboxylic acid (TCA) cycle is a central metabolic hub that generates acetyl-CoA, a HAT substrate for histone acetylation. ATP citrate lyase (ACLY) converts the TCA cycle metabolite citrate to acetyl-CoA. We traced carbons from ^13^C glucose into TCA cycle metabolites in WT and *Odc*^−/−^ T_H_ cells. Although glucose was metabolized with largely equal efficiency to pyruvate and citrate in WT and *Odc*^−/−^ T_H_ cells, there was a significant reduction in glucose carbons found in α-ketoglutarate, 2-HG, and succinate—metabolites synthesized after citrate in the TCA cycle (Figure 7G). Glucose-derived carbons in TCA metabolites post-citrate were also decreased in activated *Dohh*^−/−^ CD4^+^ T cells (Figure 7H).

We reasoned that if the TCA cycle of *Odc*^−/−^ and *Dohh*^−/−^ T cells was perturbed at citrate, we might see anaplerotic replenishment of the cycle through glutamine catabolism to α-ketoglutarate, a step immediately after citrate. The contribution of carbons from ^13^C glutamine to TCA metabolites was increased in *Odc*^−/−^ T_H_ subsets (Figure S7J), and activated *Dohh*^−/−^ T cells (Figures S7K). These data suggest loss of the polyamine-hypusine axis remodels the TCA cycle, with decreased glucose oxidation post-citrate and enhanced fueling of the cycle by glutamine.

Next, we questioned if this altered TCA cycle, was due to preferential citrate metabolism to acetyl-CoA, rather than the next step in the TCA cycle. As acetyl-CoA is a HAT substrate, we pondered if increased citrate flux to acetyl-CoA accounted for the defective T_H_ lineage commitment in polyamine-deficient T cells that our data suggested was due to excessive histone acetylation. We exposed WT and *Odc*^−/−^ T_H_17 cells to the ACLY inhibitor BMS303141. ACLY inhibition restored the aberrant IFN-γ observed in *Odc*^−/−^ T_H_17 cells back to WT levels (Figure 7I). Genetically deleting *Acly* in WT and *Odc*^−/−^ T_H_17 cells with *Acly* guides (g*Acly*) reduced ACLY protein compared to gCTRL cells (Figure 7J), and *Odc*^−/−^ T_H_17 cells lacking ACLY displayed reduced IFN-γ and T-bet, indicative of corrected lineage commitment (Figure 7K). These observations intimate that loss of polyamine metabolism, and with it hypusine synthesis, rewires the TCA cycle, likely increasing citrate flux to acetyl-CoA, helping to drive increased histone acetylation by HATs. The ultimate consequence of this is substantial transformation of the chromatin landscape that precludes T_H_ cells from accurate lineage selection during differentiation.

## Discussion

We show here that polyamine metabolism is fundamental for faithful T_H_ lineage commitment and this operates through spermidine acting as a substrate for eIF5A hypusination. This work agrees with recent reports that implicate polyamines in supporting CD4^+^ T cell effector programs (Carriche et al., 2021; Wu et al., 2020). We show that the polyamine-hypusine axis directs T_H_ lineage commitment by ensuring the correct chromatin configuration is in place for T cell specification. T cells employ multiple epigenetic mechanisms to maintain accurate T_H_ lineage commitment (Kanno et al., 2012; Wilson et al., 2009), and our study places polyamine metabolism as a critical regulator of the T cell epigenome. Similarly, Odc deletion in macrophages also results in increased inflammation due to altered histone modification (Hardbower et al., 2017).

We report that histone acetylation is key to how polyamine metabolism controls the epigenome. Increased histone acetylation occurs at the IFN-γ and IL-4 loci during T_H_1 and T_H_2 differentiation, respectively. TCR signaling leads to initial remodeling and locus opening in a cytokine-independent fashion. Subsequently, cytokine signaling reinforces polarization by expanding and maintaining accessibility at relevant cytokine loci (Fields et al., 2002). Our ATAC-seq experiments investigating chromatin accessibility suggest that polyamine metabolism governs this initial antigen-driven remodeling. In *Odc*^−/−^ and *Dohh*^−/−^ CD4^+^ T_H_ cells this TCR-driven refashioning of the epigenome becomes flawed due to dysregulated histone acetylation. This is highlighted in *Odc*^−/−^ and *Dohh*^−/−^ T_H_0 cells in which lineage-specific TFs and cytokines are expressed merely in response to antigen stimulation. Although pharmacological or genetic ablation of specific HATs rescued many facets of T_H_ differentiation in the absence of polyamine metabolism, it was impossible to restore IL-17 expression in T_H_17 cells by modulating histone acetylation. This suggests that although the polyamine-hypusine axis controls the expression of numerous immune loci via the epigenome, its influence on IL-17 synthesis is through a different mechanism. eIF5A is a translation elongation factor assisting the translation of specific transcripts (Gutierrez et al., 2013; Pelechano and Alepuz, 2017; Schuller et al., 2017). eIF5A may be involved directly in IL-17 transcript translation, possibly explaining why reducing histone acetylation in polyamine- or hypusine-deficient T_H_17 cells is insufficient to restore IL-17 expression.

Dysregulated expression of non-canonical cytokines in all *Odc*^−/−^ and *Dohh*^−/−^ T_H_ subsets suggests that the cross-inhibitory mechanisms that govern T_H_ lineage fidelity are obsolete when polyamines are limiting. We believe this adds further support to a core role for polyamine metabolism in regulating the T cell epigenetic landscape. *In vivo*, CD4^+^ T cells can adopt features of multiple T_H_ lineages simultaneously (e.g., T_H_1 cytokine expression was observed in T_H_17 cells and T_regs_ in disease) (Dominguez-Villar et al., 2011; Hirota et al., 2011), and conversion of T_regs_ into pathogenic T_H_17 cells has been described in patients with autoimmune arthritis (Komatsu et al., 2014). T-bet^+^ Gata-3^+^ CD4^+^ T cells can occur in parasite infection (Hegazy et al., 2010), and T-bet/RORγt double-positive cells have been seen in murine experimental autoimmune encephalomyelitis models (Ghoreschi et al., 2010; Nistala et al., 2010). Because we show here that polyamine metabolism limits ectopic expression of lineage-defining TFs and cytokines, restraints on polyamine synthesis or bioavailability in disease settings may promote these hybrid T cell populations that are often associated with pathogenesis. Indeed, this lack of T_H_ lineage focus in *Dohh-* and *Dhps*-deficient T cells is sufficient to cause fatal inflammation in *Dohh*-ΔT and *Dhps*-ΔT mice. Because CD4-cre deletes in all cells expressing *Cd4* during development, CD8^+^ T cells were also deficient in the polyamine-hypusine axis in our studies, and in initial analyses, these cells also exhibited dysregulated gene expression (data not shown). The contribution of these cells to the disease states described here remains to be determined. However, the colitis observed here in *Dohh*-ΔT and *Dhps*-ΔT mice is believed to be primarily CD4^+^ T cell-mediated (Shale et al., 2013), and in the *Odc*^−/−^ T cell transfer model purified CD4^+^ T cells alone accelerated colitis. Colitis manifested strongly in the hypusine-deficient mice, but was only conferred by T cell transfer in the ODC-deficient setting. T cells in *Odc*-ΔT mice may still acquire polyamines from the environment for hypusine synthesis, whereas *Dohh*- and *Dhps*-deficient T cells cannot make hypusine, even if polyamine substrates are present.

We propose that control of T_H_ lineage fidelity by polyamine metabolism via the epigenome is ultimately driven by TCA cycle regulation. TCA cycle metabolites can impact the epigenome and T cell differentiation (Tyrakis et al., 2016). We previously found the polyamine-hypusine pathway to be critical for TCA cycle integrity by maintaining mitochondrial and TCA cycle enzyme expression. Specifically, certain transcripts contained mitochondrial-targeting sequences that were hyper-dependent on eIF5A^H^ for efficient translation. We identified perturbed TCA cycle flux after acute polyamine depletion or DHPS inhibition (Puleston et al., 2019). In our current study, using models of chronic gene deletion of *Odc*, *Dohh*, and *Dhps* in T cells, we observed similarly perturbed TCA cycle metabolism. Overall, a picture emerges that polyamines and hypusine have specific effects on mitochondria, but determining how this regulation occurs requires further study. Functionally, eIF5A is a translation factor that when hypusinated preferentially regulates the translation of transcripts with specific sequence properties (Gutierrez et al., 2013; Pelechano and Alepuz, 2017; Schuller et al., 2017). The specific transcripts regulated by eIF5A in differentiating CD4^+^ T cells, and how their translation might impact the chromatin and transcriptional states of these cells, is a target of future research.

Given the established role of polyamines in controlling cell cycle (Wang et al., 2011), we place this pathway as a central process coupling T cell proliferation and differentiation. Due to the ubiquity of polyamines, these ideas may extend beyond T cells—where polyamine metabolism could be crucial for supporting lineage selection across many cell types and may be of particular importance in stem cells where cell division and differentiation are robustly linked.

### Limitations of study

Our study demonstrates a role for the polyamine-hypusine axis in supporting CD4^+^ T_H_ differentiation through epigenome regulation. We suggest this may arise through TCA cycle perturbations. However, how eIF5A^H^ ultimately maintains an intact TCA cycle in T cells remains to be determined. It is also unclear if increased acetyl-coA drives the enhanced histone acetylation that we believe leads to chromatin remodeling or whether altered HAT expression and/or activity also contribute. Our understanding of which specific transcripts are eIF5A-dependent in T cells remains unclear.

## STAR★Methods

### Key resources table

**Table:** 

REAGENT or RESOURCE	SOURCE	IDENTIFIER
**Antibodies**		

Anti-mouse CD4	Biolegend	Clone 53-6.7
Anti-mouse CD44	Biolegend	Clone IM7
Anti-mouse CD45.1	Biolegend	Clone A20
Anti-mouse CD45.2	Biolegend	Clone 104
Anti-mouse TCRβ	Biolegend	Clone H57-597
Anti-mouse CD25	Biolegend	Clone 3C7
Anti-mouse CD45	Biolegend	Clone 30-F11
Anti-mouse CD62L	Biolegend	Clone MEL-14
Anti-mouse IL-17A	Biolegend	Clone TC11-18H10.1
Anti-mouse IL-17F	Biolegend	Clone 9D3.1C8
Anti-mouse IFN-γ	Biolegend	Clone XMG1.2
Anti-mouse IL-5	Biolegend	Clone TRFK5
Anti-mouse IL-13	Invitrogen	Clone eBio13A
Anti-mouse T-Bet	Biolegend	Clone 4B10
Anti-mouse GATA-3	Biolegend	Clone 16E10A23
Anti-mouse RORγt	BD Biosciences	Clone Q31-378
Anti-mouse FoxP3	Biolegend	Clone MF-14
H3k9Ac	Cell Signaling	Clone C5B11
H2k27Ac	Cell Signaling	Clone D5E4
H3k4me3	Cell Signaling	Clone C42D8
H3k27Me3	Cell Signaling	Clone C36B11
H3k36Me3	Abcam	Cat: ab9050
Anti-mouse ornithine decarboxylase	Abcam	Cat: ab97395
Anti-mouse spermidine synthase	Abcam	Cat: ab241496
Anti-mouse spermine synthase	Abcam	Cat: ab248996
Anti-mouse GAPDH	Cell Signaling	Clone: D16H11
Anti-mouse β-actin	Abcam	Cat: ab8226
Anti-mouse α-tubulin	Abcam	Cat: ab7291
Anti-mouse/human eIF5A	BD Bioscience	Clone 26/eIF-5a
Anti-mouse/human hypusine	Millipore	Cat: ABS1064
Anti-mouse deoxyhypusine synthase	Abcam	Cat: ab190266
Anti-mouse deoxyhypusine hydroxylase	Abcam	Cat: ab122946
Anti-mouse KAT2A (GCN5L2)	Cell Signaling	Clone C26A10
Anti-human KAT3B (P300)	Abcam	Cat: ab54984
Anti-mouse ACLY	Cell Signaling	Clone D1X6P
Anti-mouse IFN-γ (neutralizing)	BioXCell	Clone XMG1.2
Anti-mouse IL-4 (neutralizing)	BioXCell	Clone 11B11
Anti-mouse IL-5 (neutralizing)	BioXCell	Clone TRFK5
Anti-mouse IL-2 (neutralizing)	BioXCell	Clone JES6-5H4
Anti-mouse IL-13 (neutralizing)	Invivogen	Clone 8H8
Anti-mouse CD3 (*in vitro* T cell activation)	BioXCell	Clone 17A2
Anti-mouse CD3 (*in vivo* anti-CD3 model)	BioXCell	Clone 145-2C11
Anti-mouse CD28	BioXCell	Clone 37.51

**Chemicals, peptides, and recombinant proteins**		

Collagenase Type VIII	Sigma	Cat. C2139
FBS	GIBCO	Lot. 1640960
Recombinant human IL-2	Peprotech	Cat. 200-02
Recombinant human TGFβ	Peprotech	Cat. 100-21
Recombinant mouse IL-12	Peprotech	Cat. 210-12
Recombinant mouse IL-4	Peprotech	Cat. 214-14
Recombinant mouse IL-1β	Peprotech	Cat. 211-11B
Recombinant mouse IL-6	Peprotech	Cat. 216-16
Recombinant mouse IL-23	Biolegend	Cat. 589002
Alt-R S.p. Cas9 Nuclease V3	IDT	Cat. 1081059
Alt-R Cas9 Electroporation Enhancer	IDT	Cat. 1075916
C646	Sigma	Cat. 382113
CPTH2	Cayman	Cat. 12086
BMS 303141	Tocris	Cat. 4609
Putrescine hydrochloride	Sigma	Cat. P5780
1,4-Butandiamine (13C4, 98%) (13C Putrescine)	Cambridge Isotope	Cat. CLM-6574
13C Glucose	Cambridge Isotope	Cat. CLM-1396
13C Glutamine	Cambridge Isotope	Cat. CLM-1822
L-methionine-15N	Sigma	Cat. 609242
13C L-proline	Cambridge Isotope	Cat. CLM-2260
13C arginine	Cambridge Isotope	Cat. CLM-2265
Methoxyamine	Santa Cruz	Cat. sc-263468
tert-butyldimethylchlorosilane	Sigma	Cat. 375934
DNase I	Sigma	Cat. 11284932001
FxCycle	Thermo	Cat. F10347
Collagenase Type IV	GIBCO	Cat. 17104019

**Critical commercial assays**		

LEGENDplex	Biolegend	Cat. 741044
RPMI 1640 media for SILAC	Thermo	Cat. 88365
RPMI, no methionine	Thermo	Cat. A1451701

**Deposited data**		

RNA-seq	Superseries: GSE157598	GEO: GSE157596
ATAC-seq	Superseries: GSE157598	GEO: GSE157597

**Experimental models: organisms/strains**		

Odc^Flox/Flox^	KOMP Repository	N/A
CD45.1 C57BL/6J mice	Jackson Labs.	#002014
Great mice	Jackson Labs.	#017581
C57BL/6J mice	Jackson Labs.	#000664
*Rag1*^*−/−*^ mice	Jackson Labs.	#002216
CD4-Cre mice	Jackson Labs.	#022071
Dohh^Flox/Flox^	Gift from S. Balabanov	N/A
Dhps^Flox/Flox^	Gift from S. Balabanov	N/A

**Oligonucleotides**		

*Dhps* gRNA #1	IDT	Mm.Cas9.DHPS.1.AA
*Dhps* gRNA #2	IDT	Mm.Cas9.DHPS.1.AE
*P300* gRNA #1	IDT	Mm.Cas9.EP300.1.AC
*P300* gRNA #2	IDT	Mm.Cas9.EP300.1.AE
*Kat2a* gRNA #1	IDT	Mm.Cas9.KAT2A.1.AA
*Kat2a* gRNA #2	IDT	Mm.Cas9.KAT2A.1.AD
*Acly* gRNA #1	IDT	Mm.Cas9.ACLY.1.AE
*Acly* gRNA #2	IDT	Mm.Cas9.ACLY.1.AF
*gCTRL* (non-specific control guide RNA)	IDT	Mm.Cas9.GCGAGGTATTCGGCTCCGCG
Alt-R CRISPR-Cas9 tracrRNA	IDT	Cat. 1072534

**Software and algorithms**		

Galaxy platform	Afgan et al., 2016	N/A
Deeptools	Ramírez et al., 2016	N/A
STAR	Dobin et al., 2013	N/A
FeatureCounts	Liao et al., 2014	N/A
DESeq2	Love et al., 2014	N/A
Morpheus	Broad Institute	N/A
DAVID	Huang et al., 2009	N/A
Trimmomatic	Bolger et al., 2014	N/A
Bowtie2	Langmead and Salzberg, 2012	N/A
SAM tools	Li et al., 2009	N/A
MACS2	Zhang et al., 2008	N/A
Bedtools	Quinlan and Hall, 2010	N/A

### Resource availability

#### Lead contact

Further information and requests for resources and reagents should be directed and will be fulfilled by the lead contact, Erika L. Pearce (pearce@ie-freiburg.mpg.de).

#### Materials availability

Mouse lines generated in this study are available upon request to the lead contact.

#### Data and code availability

The sequencing datasets produced in this study are deposited under SuperSeries GSE157598 (RNA-Seq ID: GSE157596; ATAC-Seq ID: GSE157597).

### Experimental model and subject details

#### Mice

Wild-type C57BL/6, Great, *Rag1*^*−/−*^, CD45.1 SJL, and mice expressing Cre recombinase (CD4Cre) under the control of the CD4 promoter, were all purchased from Jackson Laboratories. *Dohh*^*flox/flox*^ and *Dhps*^*flox/flox*^ were a gift from Stefan Balabanov, Zurich. *Odc*^*flox/flox*^ mice were purchased from KOMP repository. All mice were bred and maintained under specific pathogen free conditions under protocols approved by the Animal Welfare Committee of the Max Planck Institute of Immunobiology and Epigenetics, Freiburg, Germany, and The St. Jude Institutional Animal Care and Use Committee, Memphis, USA, in accordance with the Guide for the Care and Use of Animals. Mice used for all experiments were littermates and matched for age and sex (both male and female mice were used). Mice for all strains were typically 6-10 weeks of age. Survival curves in Freiburg and Memphis were plotted using the time post-birth that mice were found with rectal prolapse, at which point mice would be sacrificed.

### Method details

#### Cell Culture

For *in vitro* culture, naive CD4^+^ T cells were isolated by negative selection using a Stem Cell kit according to the manufacturer’s instructions (Stem Cell, Cat: 19765). Unless otherwise stated, naive CD4^+^ T cells were isolated from spleen and lymph nodes. Cells were cultured in RPMI 1640 media supplemented with 10% FCS, 2 mM L-glutamine, 100 U/mL penicillin/streptomycin and 55 μM β-mercaptoethanol in 48 well plates at a seeding concentration of 5x10^5^ cells per well. All activations were done with 5 ug/mL anti-CD3, 2 ug/mL anti-CD28, and 100 U/mL IL-2. T_H_ polarizations were performed as follows: T_H_0 – 10 ng/mL IL-2, 10 μg/mL anti-IFN-γ, 10 μg/mL anti-IL-4. T_H_1 – 4 μg/mL anti-IL-4, 10 ng/mL IL-12, 10 ng/mL IL-2. T_H_2 – 4 μg/mL anti-IFN-γ, 10 ng/mL IL-4, 10 ng/mL IL-2. T_H_17 – 10 μg/mL anti-IFN-γ, 10 μg/mL anti-IL-4, 5 ng/mL IL-6, 5 ng/mL TGF-β, 10 μg/mL IL-1β. T_reg_ – 4 μg/ mL anti-IFN-γ, 4 μg/mL anti-IL-4, 10 ng/mL TGF-β, 10 ng/mL IL-2. Cells were analyzed on day 4 of polarization. Other blocking antibodies used were anti-IL-2, anti-IL-5 (both BioXcell) and anti-IL-13 (Invivogen). For the ^13^C arginine tracing under different cytokine conditions in Figures 1J and S1C, cytokines and blocking antibodies were used at the following concentrations IL-2 (10 ng/mL), IL-4 (10 ng/mL), IL-12 (10 ng/mL), IL-6 (10 ng/mL), TGF-β (5 ng/mL), IL-1β (10 μg/mL), IL-23 (20 ng/mL, Biolegend), anti-IL-4 (10 μg/mL), and anti-IFN-γ (10 μg/mL). For the cytokine blocking experiments in(Figures S1I and S3D, antibodies were used at the following concentrations: anti-IL-2, anti-IL-4, anti-IL-5, anti-IFN-γ (low = 10 μg/mL, medium = 20 μg/mL, high = 50 μg/mL) and anti-IL-13 (low = 1 μg/mL, medium = 2 μg/mL, high = 5 μg/mL). All cytokines were from Peprotech and all blocking antibodies were from BioXcell, unless otherwise stated. The following drug treatments were used 20 μM C646, 250 μM putrescine hydrochloride (all Sigma), 20 μM CPTH2 (Cayman), 30 μM BMS303141 (Tocris).

#### Western blot

For western blot analysis, cells were washed with ice cold PBS and lysed in 1 x Cell Signaling lysis buffer (20 mM Tris-HCl, [pH 7.5], 150 mM NaCl, 1 mM Na_2_EDTA, 1 mM EGTA, 1% Triton X-100, 2.5 mM sodium pyrophosphate, 1 mM β- glycerophosphate, 1 mM Na_3_VO_4_, 1 μg/mL leupeptin (Cell Signaling Technologies), supplemented with 1 mM PMSF. Samples were frozen and thawed 3 times followed by centrifugation at 20,000 x g for 10 min at 4°C. Cleared protein lysate was denatured with LDS loading buffer for 10 min at 70°C, and loaded on precast 4% to 12% bis-tris protein gels (Life Technologies). Proteins were transferred onto nitrocellulose membranes using the iBLOT 2 system (Life Technologies) following the manufacturer’s protocols. Membranes were blocked with 5% w/v milk and 0.1% Tween-20 in TBS and incubated with the appropriate antibodies in 5% w/v BSA in TBS with 0.1% Tween-20 overnight at 4°C. All primary antibody incubations were followed by incubation with secondary HRP-conjugated antibody (Pierce) in 5% milk and 0.1% Tween-20 in TBS and visualized using SuperSignal West Pico or femto Chemiluminescent Substrate (Pierce) on Biomax MR film (Kodak). Antibodies used: anti-ODC, anti-DHPS, anti-DOHH, anti-β-Actin, anti-KAT3B (P300), α-tubulin (Abcam), anti-GAPDH, anti-KAT2A (GCN5), anti-ACLY (Cell Signaling), ant-EIF5A (BD Bioscience), anti-hypusine (Millipore).

#### Metabolomics

##### Metabolite Tracing

With the exception of ^13^C putrescine tracing, in which cells were treated with ^13^C putrescine for the final 48 hours of culture, tracing of all heavy-labeled substrates was performed by placing cells into fresh culture media containing heavy labeled substrate with fresh cytokines and blocking antibodies for the final 24 hours of culture. All tracing culture media was also supplemented with 10% dialyzed FCS, with the exception of ^13^C putrescine tracing. ^13^C-Argining tracing was performed by culturing cells in SILAC media supplemented with 0.2 mM L-lysine and 1.1 mM ^13^C-Arginine (Cambridge Isotope Lab.). ^13^C glutamine tracing was carried out by culturing cells in glutamine-free RPMI supplemented with 4 mM ^13^C glutamine (Cambridge Isotope Lab.). ^13^C glucose tracing was carried out in glucose-free RPMI with 11 mM ^13^C glucose (Cambridge Isotope Lab.). For ^13^C proline tracing, cells were cultured in DMEM (that lacks L-proline) with 0.2 mM ^13^C proline (Cambridge Isotope Lab.). ^15^N Methionine tracing was performed in methionine-free RPMI supplemented with 0.1 mM ^15^N methionine (Sigma). ^13^C putrescine (Cambridge Isotope Lab.) tracing took place in RPMI.

For harvest, cells were rinsed with cold PBS and metabolites extracted using a mix of cold methanol, acetonitrile and water (50:30:20) kept on dry ice. For polyamine detection, metabolites were extracted in this same mix but with 1.5% hydrochloric acid. Following mixing and centrifugation, the supernatant was collected and dried via centrifugal evaporation. Dried metabolite extracts were resuspended in pyridine and derivatized with methoxyamine (sc-263468 Santa Cruz Bio) for 60 minutes at 37°C and subsequently with N-(tert-butyldimethylsilyl)-N-methyl-trifluoroacetamid, with 1% tert-butyldimethylchlorosilane (375934 Sigma-Aldrich) for 30 minutes at 80°C. Isotopomer distributions were measured using a DB5-MS GC column in a 7890 GC system (Agilent Technologies) combined with a 5977 MS system (Agilent Technologies). Correction for natural isotope abundance and calculation of fractional contribution was performed as described elsewhere (Buescher et al., 2015).

##### Polyamine Quantification

Metabolites were quantified by LC-MS using HILIC Chromatography on an Acquity UPLC BEH Amide column 1.7 μm, 2.1x100 mm on a 1290 Infinity II UHPLC system (Agilent Technologies) combined with targeted detection in a 6495 MS system (Agilent Technologies). Peak areas were normalized to ^13^C labeled internal standard (ISOtopic Solutions). Polyamines were extracted from CD4^+^ T cells using a mix of cold methanol, acetonitrile and water (50:30:20) containing 1.5% hydrochloric acid.

For tracing of heavy-labeled substrates into polyamines, cells were washed with ice-cold PBS and metabolites were extracted using extraction buffer comprising 50:30:20 methanol:acetonitrile:water (plus 2% acid for polyamine extraction) cooled on dry ice for 30 minutes beforehand. Samples were centrifuged at maximum speed for 10 minutes to remove protein debris and supernatants were dried using a Genevac EZ2 speed vac and then stored at −80 until acquisition. Metabolite analysis was carried out using an Agilent 1290 Infinity II UHPLC in line with a Bruker Impact II QTOF (resolution > 50,000 FSR). Details of LC-MS methods are described by Edwards-Hicks et al. (2020). In brief, chromatographic separation of metabolites was performed using a Phenomenex Luna NH2 column (50 × 2 mm, 3 μm particles) using a solvent gradient of 100% buffer B (5 mM ammonium carbonate in 90% acetonitrile) to 90% buffer A (10 mM NH4 in water). The mass spectrometer was operated in negative mode. Chromatographic separation of polyamines was performed using a Waters CSH C18 column (100 × 2 mm, 1.7 μm particles) using a solvent gradient of 100% buffer A (0.1% formic acid in water) to 97% buffer B (50:50 acetonitrile:methanol). The mass spectrometer was operated in positive mode. Data analysis was performed using AssayR software (Wills et al., 2017). Metabolites were identified by matching accurate mass and retention time to known standards.

#### Flow Cytometry

Flow cytometric staining was performed as previously described (Chang et al., 2015). To assess cytokine staining *in vitro* and *ex vivo*, cells were re-stimulated with 50 ng/mL PMA and 1 μg/mL ionomycin in the presence of brefeldin A (Biolegend) for 5 hours. Intracellular cytokine staining was performed using BD CytoFix/CytoPerm kit (BD Biosciences) and nuclear staining of transcription factors using the FoxP3 Permeabilisation kit (eBioscience). Cells were stained with Live/Dead viability dye (Thermo) prior to antibody staining. Cells were collected on LSR II and Fortessa flow cytometers (BD Biosciences) and analyzed using FlowJo (TreeStar) software. The following antibodies were used: anti-CD4, anti-TCRβ, anti-CD45, anti-IL17A, anti-IL-17B, anti-IFN-γ, anti-T-bet, anti-GATA-3, anti-FoxP3 (all Biolegend), anti-RORγt (BD Bioscience), anti-ODC, anti-spermine synthase (both Abcam). For analysis of chromatin marks, cells were gated on Ki-67^+^ cells and diploid cells with ‘single’ DNA content based on FxCycle (dapi, Thermo) staining in the live cell gate. Primary antibodies were stained for 90 minutes in permeabilization buffer at room temperature, followed by staining with the relevant secondary antibody for 30 minutes. The following antibodies were used: anti-H3k4Me^3^, anti-H3k9Ac, anti-H3k27Ac, anti-H3k9Me^3^ (all Cell Signaling), and anti-H3k36Me^3^ (Abcam).

#### T cell transfer colitis

4x10^5^ naive CD4^+^ T cells (CD45Rb^hi^ CD25^-^ CD44^lo^ CD62L^+^) from WT or *Odc*-ΔT mice were adoptively transferred into 12 week old *Rag1*^*−/−*^ recipient mice (Jackson) by intravenous injection. For experiments involving the co-transfer of naive CD4^+^ T cells with or without T_regs_, we transferred 4x10^5^ naive CD4^+^ T cells (CD45Rb^hi^ CD25^-^ CD44^lo^ CD62L^+^) from CD45.1 SJL mice with or without 1.5x10^5^ T_regs_ (CD4^+^ CD25^+^ sorted by FACS post-CD4^+^ T cell enrichment) from either *Dohh*-ΔT mice or their WT littermate controls. Disease score was calculated at the experimental endpoint using the following criteria:

**Table:** 

Score	Weight loss	Stool Consistency	Blood in stool
*0*	None	Normal	Negative
*1*	1-5%	Soft but still formed	Negative
*2*	6-10%	Soft	Blood traces in stool visible
*3*	11-18%	Very soft; wet	Blood traces in stool visible
*4*	> 18%	Watery diarrhea	Gross rectal bleeding

#### *In vivo* treatment with anti-CD3 monoclonal antibody

Mice were injected intraperitoneally with CD3-specific antibody (clone 2C11, BioXcell, 50 μg/mouse) three times with 48 hours between each injection. Mice were sacrificed for analysis 4 hours after the third injection.

#### Cytokine measurement in serum and supernatent

Serum and supernatent cytokine were measured by cytokine bead array using the LEGENDPlex Th cytokine panel according to the manufacturer’s instructions (Biolegend) on a BD Fortessa flow cytometer (BD Biosciences).

#### Isolation of CD4^+^ T cells from Non-Lymphoid Tissues

For lung, mice were perfused with PBS through the left ventricle. Organs were cut up into 1-3 mm^3^ pieces and digested in 1 ug/mL Collagenase A (Sigma) and 0.5 mg/mL Dnase I (Roche) in RPMI at 37°C for 30 minutes on a shaker. Digested organs were then mechanically disrupted through a cell strainer prior to flow cytometry staining. Cell suspensions from the colonic lamina propria were prepared as follows: briefly, colons were isolated, cleaned, cut into small pieces and placed in RPMI/5% FCS supplemented with 5 mM EDTA. Tubes were then placed in 37°C in a shaking incubator to remove intestinal epithelial cells (IECs). This washing process was repeated twice, followed by one incubation with RPMI/5% FCS containing 15 mM HEPES. Digestion of the colon tissue was performed in RPMI/0.5% FCS containing 1 mg/ml type VIII collagenase (Sigma Aldrich) and 40 μg/ml DNase I (Roche) at 37°C for 60 minutes on a shaker. Supernatants were then filtered and a three-layered discontinuous Percoll gradient was used to obtain an enriched leukocyte fraction.

For the isolation of cells from the small intestine lamina propria – small intestines were isolated, cleaned and cut into 2cm pieces. Tissue was then incubated for 25 minutes in RPMI/3% FCS supplemented with 5mM EDTA and 0.15 mg/ml DTT at 37°C with shaking. After, small intestines were washed 3 times with RPMI containing 2mM EDTA. Tissue was then digested for 30 minutes in RPMI containing 0.1mg/ml Liberase TL (Roche) and 50ug/ml DNase I (Roche) at 37°C with shaking. After that a three-layered discontinuous Percoll gradient was used to enriched for the leukocyte fraction.

#### Histology

Samples of the colon and caecum were collected and fixed in buffered 10% of 36% formalin solution for 24 hours and then stored in 70% ethanol prior to processing. Haematoxylin and eosin (H&E) staining was performed on 4–5 mm paraffin-embedded sections.

#### RNA sequencing analysis

RNA was extracted using the RNeasy Kit (QIAGEN) according to manufacturer instructions and quantified using Qubit 2.0 (Thermo Fisher Scientific) following the manufacturer’s instructions. Libraries were prepared using the TruSeq stranded mRNA kit (Illumina) and sequenced in a HISeq 3000 (Illumina) by the Deep-sequencing Facility at the Max-Planck-Institute for Immunobiology and Epigenetics. Sequenced libraries were processed with deepTools (Ramírez et al., 2016), using STAR (Dobin et al., 2013), for trimming and mapping, and featureCounts (Liao et al., 2014) to quantify mapped reads. Raw mapped reads were processed in R (Lucent Technologies) with DESeq2 (Love et al., 2014) to generate normalized read counts to visualize as heatmaps using Morpheus (Broad Institute) and determine differentially expressed genes with greater than 2 fold change and lower than 0.05 adjusted p value. Gene ontology analysis was performed used the free online platform DAVID (Huang et al., 2009) and Ingenuity® pathway Analysis (QIAGEN). Supervised clustering of gene expression was performed with pheatmap (version2012) using Ward’s minimum variance method (Murtagh and Legendre, 2014).

#### ATAC sequencing analysis

Libraries were prepared using the Nextera DNA library Prep Kit (Illumina) adapting a published protocol (Buenrostro et al., 2015). Briefly, 5x10^4^ T cells treated as described were washed in PBS and then lysed in 10 mM Tris-HCl, pH 7.4,10 mM NaCl, 3 mM MgCl_2_ and 0.1% Igepal CA-630 (all Sigma). Nuclei were then spun down and then resuspend in 25 μL TD (2x reaction buffer), 2.5 μL TDE1 (Nextera Tn5 Transposase) and 22.5 μL nuclease-free water, incubated for 30 min at 37°C. DNA was purified with the QIAGEN MinElute PCR Purification Kit (Thermo Fisher Scientific). PCR amplification was performed with the NEBNext High-Fidelity 2x PCR Master Mix (New England Labs) using custom Nextera PCR Primers containing barcodes. Adaptors were removed with AMPure XP beads according to manufacturer’s protocol. Libraries were quantified with the Qubit and submitted for sequencing with a HISeq 3000 (Illumina) by the staff at the Deep-sequencing Facility at the Max-Planck-Institute for Immunobiology and Epigenetics. Sequenced samples were trimmed with Trimmomatic ^9^ and mapped using Bowtie2 (Langmead and Salzberg, 2012). Open chromatin was detected with MACS2 (Zhang et al., 2008), while differences between treatments was determined using DiffBind (Ross-Innes et al., 2012) with at least 2 fold change in accessibility and a false discovery rate lower than 0.05. For visualization only, replicate mapped files were merged with SAM tools ^13^ and coverage files were generated with deepTools and visualized alongside coverage files on IGV (Robinson et al., 2011). Bed files were analyzed with Bedtools ^15^.

#### CRISPR-Cas9

All guide RNAs were purchased from IDT. Duplexes of two separate guides per target gene were prepared by annealing (5 min; 98°C) equimolar concentrations of CRISPR gRNA and trRNA, then incubation (20 min; RT) with Alt-R S.p. Cas9 Nuclease V3 (IDT). For the delivery of RNP complexes, naive CD4^+^ T cells were washed in PBS, mixed with the RNP complexes and Electroporation Enhancer in P4 Primary Cell buffer (Lonza) immediately prior to electroporation (4D-Nucleofector; Lonza – Program DS137). Per electroporation, 60 pmol of Cas9 Nuclease and 180 pmol of annealed gRNAs were used. Electroporated cells were recovered in T cell medium for 2 hours prior to activation with αCD3/CD28 and pertinent polarizing cytokines. The following guides were used: *Mm.Cas9.Tbx21.1AC* and *Mm.Cas9.Tbx21.1AD* (T-Bet); *Mm.Cas9.EP300.1AC* and *Mm.Cas9.EP300.1AE* (P300); *Mm.Cas9.KAT2A.1AA* and *Mm.Cas9.KAT2A.1AD* (KAT2A); *Mm.Cas9.ACLY.1AE* and *Mm.Cas9.ACLY.1AF* (ACLY); *Mm.Cas9.GCGAGGTATTCGGCTCCGCG* (Non-specific control guide).

### Quantification and statistical analysis

p values were determined using unpaired Student’s t test unless otherwise stated. Differences were considered statistically significant when p < 0.05 (^∗^ < p0.05, ^∗∗^p < 0.01, ^∗∗∗^p < 0.001). Data are shown as mean ± s.e.m. Statistics were calculated using GraphPad Prism 6 software.
